# Long-range temporal correlations in neural narrowband time-series arise due to critical dynamics

**DOI:** 10.1371/journal.pone.0175628

**Published:** 2017-05-04

**Authors:** Duncan A. J. Blythe, Vadim V. Nikulin

**Affiliations:** 1 Zalando Research, Zalando SE, Berlin, Germany; 2 Neurophysics group, Department of Neurology, Charité Medical University, Berlin, Germany; 3 Department of Neurology, Max Planck Institute for Human Cognitive and Brain Sciences, Leipzig, Germany; 4 Center for Cognition and Decision Making, National Research University Higher School of Economics, Moscow, Russian Federation; University of Michigan, UNITED STATES

## Abstract

We show theoretically that the hypothesis of criticality as a theory of long-range fluctuation in the human brain may be distinguished from the theory of passive filtering on the basis of macroscopic neuronal signals such as the electroencephalogram, using novel theory of narrowband amplitude time-series at criticality. Our theory predicts the division of critical activity into *meta-universality classes*. As a consequence our analysis shows that experimental electroencephalography data favours the hypothesis of criticality in the human brain.

## Introduction

In a spatially extended physical system of homogeneous interacting elements, *critical power-law avalanche dynamics* (CD) occur when the size and lifetime of a burst of activity after a perturbation (an ‘avalanche’) follow power-law distributions [1, 2]. Theoretical approaches to CD assume a separation of time-scales [1, 3]; the model system under study is allowed to relax to equilibrium completely after a perturbation before the effect of a subsequent perturbation is considered.

However, in experiments involving real-world physical systems, individual avalanches are often not directly observable—the signal is in many cases continuous. Perturbations are generated by the presence of stochasticity intrinsic to the system and may occur at random before the termination of an avalanche caused by a prior perturbation. Thus avalanches ‘overlap’ in the temporal domain and the degree of overlap depends on the size of the system, assuming that perturbations occur at random in each component element; such overlapping generates a continuous signal.

In neuroscience, several experiments [4–6] have provided evidence *in vitro* that small isolated neuronal networks display CD; however, the networks studied do not typically display a separation between periods of inactivity and activity [5, 7]. In such experiments, a period of inactivity is defined by the network returning to a quiescent baseline level of lesser activity set by a threshold [5, 7, 8]. While this technique may work for small networks isolated in cultures, results have been contradictory *in vivo* [9–11]; no consideration has yet been given to the hypothesis that this discrepancy is due to the absence of separation of time-scales in the *continuous* neuronal activity.

When testing the hypothesis of criticality in the human brain, the problem of a lack of separation between avalanches is especially pressing. The use of invasive electrodes is problematic and research is often limited to macroscopic imaging of the entire brain, using, for instance, electroencephalographic (EEG) or magnetoencephalographic (MEG) recordings. The entire human brain never attains a quiescent level of baseline activity; the brain is continuously active rather than being relatively inactive between bursts of activity [12]. Only a few papers have claimed to test the hypothesis of criticality from the large-scale brain signals of awake human subjects [13–15]. These analyses are subject to several difficulties; firstly, the threshold set for separation between activity and inactivity is high, thus making the relationship to genuine separation of time-scales unclear. Secondly, as a consequence of this high threshold, the authors find CD only over short time-scales (<1 second). Thus no explanation may be offered on this basis of the long-term variability of human brain activity.

Such difficulties stemming from the lack of separation between activity and inactivity *in vivo* make unclear whether the results of *in vitro* experiments confirming criticality in animals [4–6] generalize to the intact brain of awake human subjects. Despite these difficulties, the question remains, why does the human brain display the same 1/*f*^*γ*^ form in its power-spectra [16] as posited by CD [3]? The most prominent alternative theory to CD proposes that the scale-free form of the power-spectra, for example, of EEG and MEG data, is due not to CD but to *passive filtering* (PF) of activity through the extracellular media [17, 18]; thus the authors show that CD is not necessary to explain the 1/*f*^*γ*^ form of the power-spectrum.

**Fig 1:**
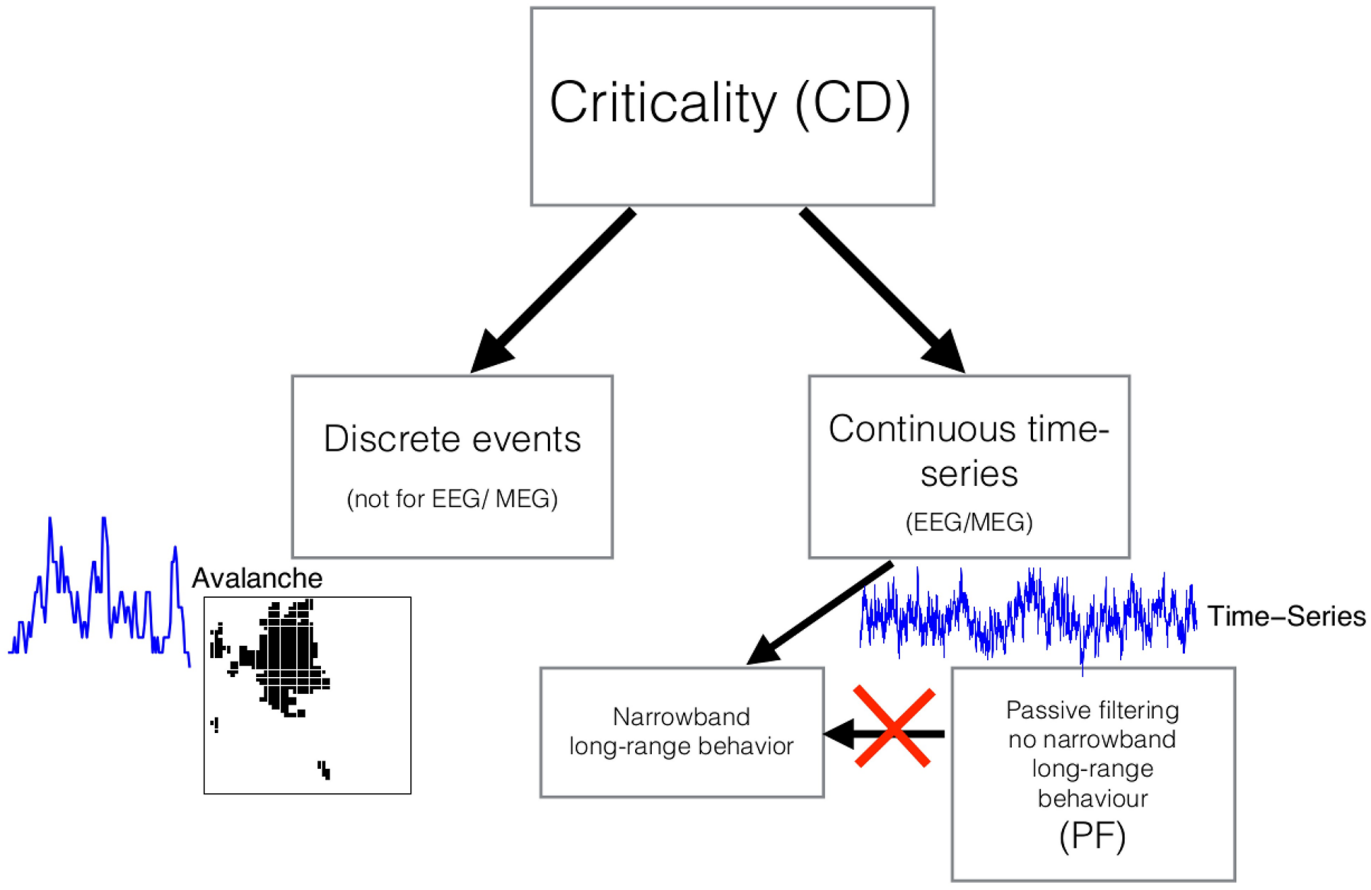
The approach taken in this paper. Criticality implies that the distibutions of *discrete* avalanches are power-law (left hand side). However analysing discrete dynamics is problematic on the basis of continuous EEG/ MEG recordings. Until now it was unclear how to distinguish criticality from alternative explanations of 1/*f*^*γ*^ power-spectra on the basis of continuous data (right hand side) such as EEG/ MEG. We show that criticality implies that the narrowbands of the continuous data have specific long-range properties (left hand branch of right hand side). The passive-filtering theory of the origin of the 1/*f*^*γ*^ form of EEG/ MEG power-spectra does not predict such long-range narrowband properties (right hand branch of right hand side). Thus we have a criterion to distinguish criticality from passive filtering on the basis of MEG/ EEG recordings. We perform this test on empirical data.

In this paper, we show that the PF explanation is insufficient in reproducing certain long-range properties of EEG data which we show theoretically must hold if the CD theory is correct. We demonstrate that if a critical system produces temporally overlapping avalanches, which superimpose linearly at the level of sensors (as is known for electrophysiological recordings [19, 20]), then the corresponding stochastic process generated by such activity may be shown to display novel properties, distinct from the form of the power-spectrum, which are *provably* not explainable by the PF theory. Our theory shows for the first time that critical universality classes naturally fall into subclasses which we refer to as *meta-universality classes*; the meta-universality classes are defined according to the spectral properties of the narrowband amplitude processes derived non-linearly from the activity produced by the system. The theory allows us to derive a test for CD in macroscopic neural recordings without requiring separation of activity and inactivity. We apply the test to EEG recordings and find that the data strongly favour the CD hypothesis over the PF hypothesis as a theory of neuronal variability over long time-scales. An illustration of our approach is displayed in Fig 1.

## Results

### Overview of theory

We assume that we record activity of a macroscopic electrophysiological signal such as the EEG or MEG and that the activity is generated by a large neural network. We assume moreover that the activity is composed of the activity of numerous local neural networks such that the total activity measured is a linear superposition of the activity of these local networks; this linear superposition is well known and studied [19]. The activity of these local neural networks is composed of bursts of activity interleaved by periods of quiescence, due to the sparseness of neuronal firing [21, 22]. In physical terms, these bursts of activity may be thought of as perturbations from equilibrium caused by the intrinsic stochasticity of the network. We shall refer to these bursts of activity as “avalanches”, independently of whether their size and lifetime are power-law distributed. Thus short bursts of activity will be referred to as “short avalanches”, despite the everyday connotation of the word “avalanche” as a “tumbling of snow of immense size”. To simplify matters we assume that at each time step a fixed number of avalanches of local neural networks are initiated. Thus the recorded signal consists of the linear superposition of all avalanches initiated at prior times which have yet to subside.

According to the CD theory the size (number of neurons firing) and lifetime (length in time) of the avalanches of local neural networks are power-law distributed [1, 2]; according to the PF theory, the size and lifetime distributions decay faster than a power-law [17, 18].

In the case of the PF theory the long range correlations/ power-law power spectrum observed in the macroscopic neural signals are generated by filtering through the extracellular medium between recording electrode and neuronal activity. The PF hypothesis asserts that this filtering is caused by ionic diffusion generated by recalibration of concentrations through the medium at the source of the neuronal activity after polarization of neuronal membranes [18].

Vital to note from the outset is that our theory predicts properties of the amplitude dynamics of the narrowband activity of macroscopic neural signals. The importance of these dynamics in the CD/ PF debate is that *they are invariant to linear filtering of the underlying signal*. This is because the signal may be considered a linear combination of its narrowband (individual frequency) activities—a linear filter has only the effect of *reweighting* these narrowbands [23]. Thus predictions made on the basis of CD regarding these narrowband dynamics circumvent the counterarguments of the PF theory.

**Fig 2:**
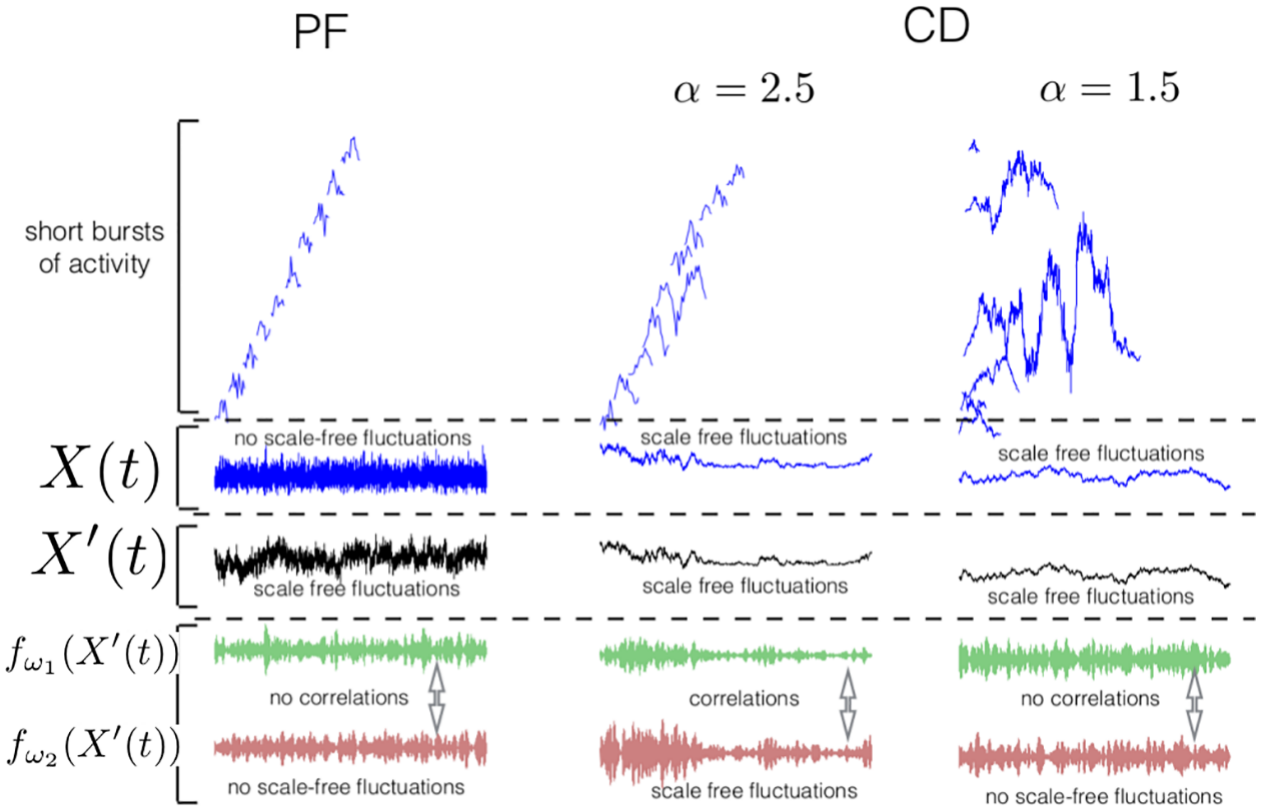
Illustration of the theory. Samples according to PF (left) and CD with the lifetime exponent *α* = 2.5, 1.5 and height exponent *β* = 1 (centre, right). Top row: avalanches *a*_*i*,*s*_(*t*) composing the continuous network signal *X*(*t*) by linear superposition (many avalanches superimposed over one another). Second row: continuous signal *X*(*t*). Third row: measured signal, filtered in the case of the PF theory (left) and a scalar multiple of the network signal *X*(*t*) in the CD case (centre, right). Fourth and fifth rows: narrowband signals at two frequencies *ω*_1_ and *ω*_2_. We observe that in all cases the observed signal *X*′(*t*) fluctuates over a range of time-scales. However the narrowband signals display pronounced fluctuations in their amplitude envelopes only in the CD model for certain exponent values. See Section: Results: Overview of Theory for a detailed description of each panel.

Fig 2 illustrates our theory. The upper row (rows delineated by the square brackets and dotted lines) displays sample avalanches which are initiated at each time-step according to both the PF and CD theories; for visualization purposes, each subsequent avalanche is displayed at a separate position in the *y*-direction (from bottom to top in time, according to when each period began). In the second row, we see the sample activity *X*(*t*), produced by the network, composed as the linear superposition (sum) of all of these avalanches. The third row displays the observed data at the measurement device *X*′(*t*), which is given according to PF by a 1/*f*^*γ*^ filtered version of *X*(*t*) but according to CD simply as a scalar multiple (dependent on the proximity to the recording electrode) of *X*(*t*). The fourth and fifth rows display narrowband time-series *f*_*ω*_1__(*X*′(*t*)), *f*_*ω*_2__(*X*′(*t*)) which are obtained from the observed activity *X*′(*t*) by linear filtering in a narrow pass-band around two distinct frequencies *ω*_1_ and *ω*_2_. In each row we visualize three cases. The left hand column corresponds to the PF theory, the right two columns correspond to two distinct universality classes in CD, which we denote by parameters *α*, *β*; the length of avalanches are distributed as *p*(*L*)∼*L*^−*α*^ and the height of avalanches as *p*(*h*)∼*L*^*β*^. Informally *α* determines how likely long avalanches are in comparison to short avalanches and *β* relates to how tall long avalanches are in comparison to short avalanches (see Section: Materials and Methods: Theoretical Results for details). In Fig 2 we consider fixed *β* since varying *β* has simply the effect of altering the results quantitatively (but not qualitatively) and controlling the exact onset of the distinct behaviours (thus we relegate detailed discussion of *β* to the theory section).

We see that the PF and CD theories make differing predictions and that the predictions of the CD theory depend on the universality class. The predictions relate to the dynamics of narrowbands *f*_*ω*_(*X*′(*t*)) for all *ω*. This holds since in both the PF and CD cases, the dynamics of *f*_*ω*_*i*__(*X*′(*t*)) are unaffected by linear filtering of *X*(*t*).

1. The PF theory predicts that *X*(*t*) is not an auto-correlated time-series over long-time scales (left, second row). This is because the avalanches do not span long enough time-scales to induce long-range correlations (left, top row). This implies that distant time-points of *X*(*t*) are independent. The long-range variability of *X*′(*t*) (where *X*′(*t*) is the observed data) is induced by passive filtering through the extra cellular medium (left, third row). This implies that the amplitude envelopes of the narrow-band time-series, *f*_*ω*_1__(*X*′(*t*)), *f*_*ω*_2__(*X*′(*t*)), are also uncorrelated over long time-scales and across frequencies (left, fourth and fifth row); this is because *f*_*ω*_*i*__(*X*′(*t*)) preserves the dynamics of *f*_*ω*_*i*__(*X*(*t*))—the process of linear filtering has no effect on narrowband dynamics.
2. For *α* = 2.5 long avalanches are far more likely than in the passive filtering case; the total variance of long avalanches outweighs the variance of small avalanches (centre, top row). This implies that the total activity of the system *X*(*t*) and therefore the observed activity *X*′(*t*) display scale-free fluctuations over all time-scales (centre, second and third rows). Because the variance of the large avalanches dominate over the small avalanches, the amplitude of the narrowband time-series display scale-free fluctuations on all time-scales; since the fluctuations arise from the swell in variance profile generated by individual avalanches which have broadband frequency content, these scale-free fluctuations are correlated across frequencies (centre, fourth and fifth rows). Informally speaking, the avalanches “protrude” from *X*′(*t*); such “protruding” causes fluctuations in all *f*_*ω*_*i*__(*X*′(*t*)).
3. For *α* = 1.5 the likelihood of long avalanches is larger than the likelihood when *α* = 2.5. This implies that the number of avalanches gradually accumulates over time (right, top). As for *α* = 2.5 the presence of long avalanches implies the presence of long-range fluctuations in *X*(*t*) and *X*′(*t*) (right, second and third rows). This is because the frequency content of the signal *X*(*t*) is given by the sum of frequency content over all active avalanches—this takes the form of a power-law. Formally, frequency content sums linearly over a superposition of avalanches, which means that whether the individual avalanches are visible or not is immaterial to whether the power-spectrum is of power-law form. However since the number of avalanches active gradually accumulates, the narrowband time-series do not display long-range correlations or correlations across frequencies (right, fourth and fifth rows). Informally, the small exponent *α* causes avalanches to “stack up” cumulatively. This process causes the profiles of the individual avalanches to become invisible, meaning that scale-free fluctuations disappear from the *f*_*ω*_*i*__(*X*′(*t*)), although *X*′(*t*) still displays scale-free fluctuations.

**Fig 3:**
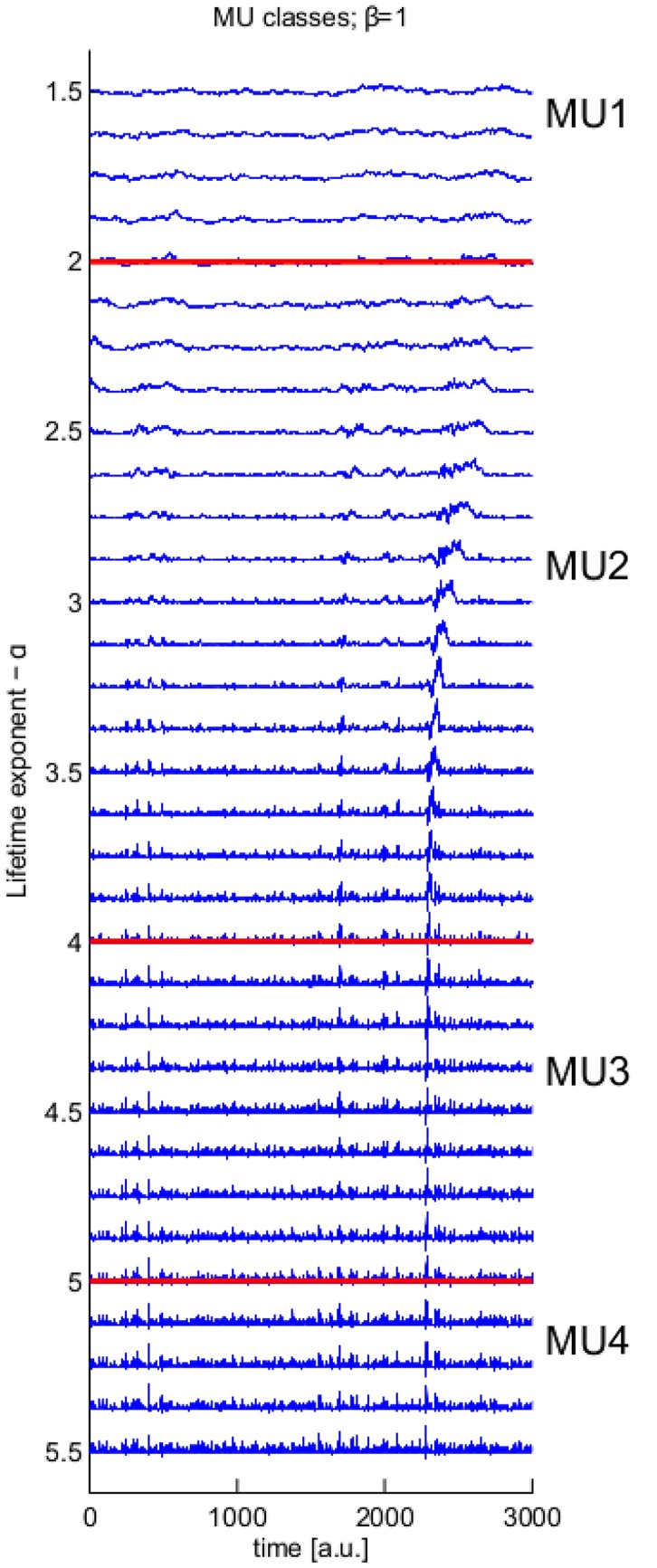
Illustration of the division of critical exponents into meta-universality classes. With *β* = 1, as the lifetime exponent *α* varies (*y*-axis), the qualitative nature of the continuous data varies, with individual avalanches only clearly visible towards the lower regions of MU2 and upper regions of MU3. See Section: Results: Overview of Theory.

See Fig 3 for an illustration of the difference between values of *α* in the case of the CD theory. As *α* decreases (from bottom to top), individual avalanches become visible in the continuous data, but for values *α* < 2, avalanches are no longer individually discernible. This continuous transition may be explained in terms of moving gradually between the three cases above: the PF theory, the CD theory with *α* = 2.5 and the CD theory with *α* = 1.5. For large values of *α*, the qualitative properties *X*(*t*) for the CD theory and the PF theory are identical; large avalanches are improbable. (lower-part of Fig 3, labelled MU4). Here the data has a homogeneous character, with individual bursts of activity not clearly visible. As *α* decreases, large avalanches become more probable, and these become discernible in the continuous time-series (middle part of Fig 3, labelled MU2-3). However, as *α* continues to decrease, the relative probability of long avalanches become so great that avalanches overlap to an extent that they are not individually discernible (top part of Fig 3).

### Theoretical results

In this section we formalize the observations made in the previous section; the formal statements are proved in Section: Materials and Methods: Theory. As a starting point we use the facts that avalanches occuring in *local* neural networks are separated by periods of inactivity, and macroscopic brain signals, such as EEG/ MEG, measure the linear superposition *X*(*t*) of activity of numerous local neural networks. That avalanches of *local* neural networks do not overlap in time on the level of small spatially local subnetworks follows from the finding that neural firing is sparse [21, 22]; note that this allows that multiple avalanches are active in the entire network (composed of all local subnetworks) at any given time; i.e. in intra-cortical experiments using multi-electrodes ranging over a couple of mm., bursts of activity are separated in time by quiescence, although when looking at the whole brain using, e.g. EEG, we see a continuous signal, which is composed of the superposition of all such spatially local avalanches. Linear superposition is well established in electrophysiology [19] and follows from the principle of superposition of electrodynamics [24] (This linear superposition was also proposed as the mechanism whereby flicker or 1/*f* noise arises from power-law dynamics in the seminal paper of [1]; although such a linear superposition may not hold for all applications it is well established in neuroelectrophysiology.) This means that the macroscopic network activity *X*(*t*) is continuous, since before the termination of any individual avalanche, several other avalanches have been ignited in other spatial areas of the network. Thus:

$$\begin{array}{c} X\left(t\right)=\sum_{s=1}^{T}\sum_{i=1}^{q_{s}}h_{s,i}a_{s,i}\left(\frac{t-s}{L_{s,i}}\right) \end{array}$$ (1)

$a_{s,i}\left(\frac{t-s}{L_{s,i}}\right)$ denotes activity at time *t* of the *i*^th^ local neural network which begins at time *s*, after a period of inactivity and lasts for *L*_*s*,*i*_ time steps. We adopt the conventions that *a*_*s*,*i*_(*t*) is normalized to have average height of 1 and is 0 for *t* ∈ (−∞, 0) ∪ (1, ∞). Thus, *h*_*s*,*i*_ denotes the average height of the time-course of activity of the *i*^th^ local neural network which begins at time *s*. To simplify matters in the following, we assume that *q*_*s*_ is constant *q*_*s*_ = *q*. All results may be extended if the *q*_*s*_ are considered as independent samples of a single random variable.

So far Eq (1) says nothing which distinguishes PF and CD. The distinction between PF and CD lies in how *h*_*s*,*i*_ and *L*_*s*,*i*_ are claimed to be distributed. According to the PF hypothesis their distributions decay faster than power-laws (see left, top row of Fig 2). For simplicity, therefore, we summarize the PF as claiming exponential distributions:

$$\begin{array}{c} p\left(L\right)∼e^{-AL} \end{array}$$ (2)

$$\begin{array}{c} p\left(h\right)∼e^{-Bh} \end{array}$$ (3)

Moreover the PF hypothesis states that power-spectra of macroscopic brain signals *X*′(*t*) are power-law because they reflect a filtered version of *X*(*t*):

$$\begin{array}{c} X^{\prime }\left(t\right)=\sum_{u=0}^{\infty }F\left(u\right)X\left(t-u\right) \end{array}$$ (4)

*F*(*u*) is a linear filter due to the extracellular media which yields a signal with power-law power spectrum from white noise input [17, 18].

Important to note is that the filter *F* leaves the amplitude dynamics of narrowbands of *X*(*t*) unaffected. This is because *F* is a linear filter, so if *c*_*ω*_ is the frequency response of *F* at the frequency *ω*, and *f*_*ω*_(*X*(*t*)) is the narrowband component of *X*(*t*) at frequency *ω* then:

$$\begin{array}{c} F\left(X\left(t\right)\right)=F(\sum_{\omega }f_{\omega }\left(X\left(t\right)\right)) \end{array}$$ (5)

$$\begin{array}{c} =\sum_{\omega }c_{\omega }f_{\omega }\left(X\left(t\right)\right) \end{array}$$ (6)

Thus the PF theory makes no claim as to the dynamics of the narrowbands, only their relative weightings in the observed signal.

On the other hand, the CD hypothesis states that macroscopic signals *X*′(*t*) reflect *X*(*t*) directly so that *X*′(*t*) = *bX*(*t*), for some scalar *b*, and that we have power law densities for *h*_*s*,*i*_ and *L*_*s*,*i*_ cut off at a value *L*_*c*_ proportional to the system size:

$$\begin{array}{ccc} p(L) & ∼ & L^{-\alpha }\\ p(h) & ∼ & L^{\beta } \end{array}$$ (7)

Notice that the power-law in *h* is described in terms of *L*. This is because CD asserts dependency between the distribution of the two quantities [3]. This dependence implies that the marginal distribution over *h* is a power-law in *h*. These power-law densities explain the power-law form of neuroelectrophysiological power-spectra [25]. In addition the CD hypothesis states that each *a*_*s*,*i*_(*t*) is an independent and identical sample from a single stochastic process, which we call *a*(*t*) [26].

As well as considering the raw measured signal X’(t) we also consider the amplitude of a narrowband linear-filtered component of *X*′(*t*), which we denote *g*_*ω*_(*X*(*t*)) (this corresponds to the amplitude envelope of oscillatory activity in the bottom row of Fig 2). See Section: Materials and Methods: Amplitudes Estimated with the Hilbert Transform for details.

**Fig 4:**
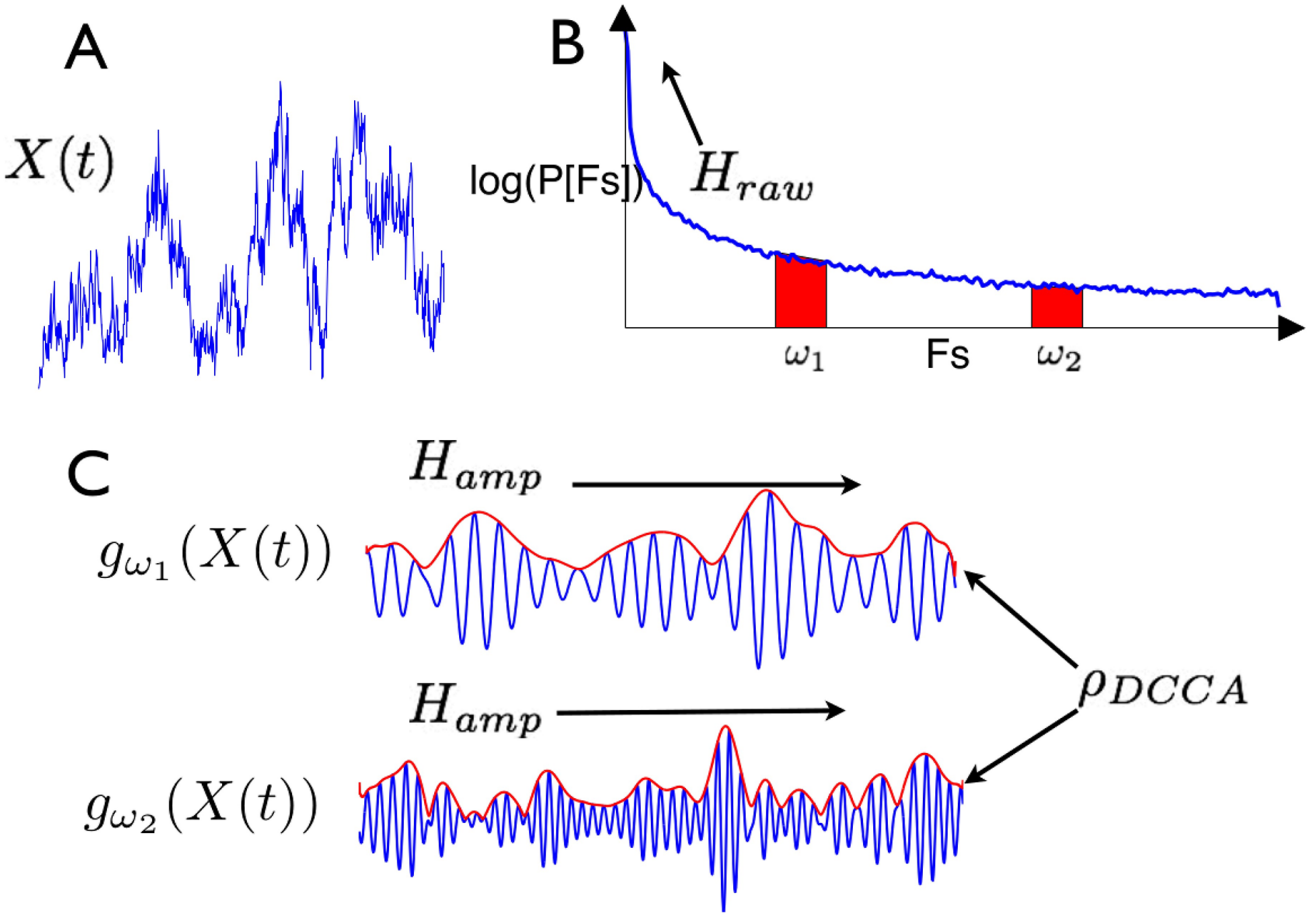
Overview of analysis steps. (A) The neural signal is extracted from the data. (B) Its power-spectum takes the form of a power-law. (C) Narrowband components in two frequency ranges (red on power-spectrum) are extracted from the signal by filtering and the amplitude envelope is extracted using the Hilbert transform (in red).

An illustration of *g*_*ω*_(*X*′(*t*)) is given in the lower-panel Fig 4; *f*_*ω*_(*X*′(*t*)) may be considered as a narrowband oscillatory signal and *g*_*ω*_(*X*′(*t*)) as the amplitude envelope of these oscillations. *g*_*ω*_(*X*′(*t*)) has been studied in many applications in neuroscience, including with regards to long range variability [27, 28].

Our theory depends on two measures of long-term variation evaluated on *g*_*ω*_(*X*(*t*)): the *Hurst exponent* and the *Detrended Cross Correlation Coefficient*. The Hurst exponent *H*, of a stationary process *Y*(*t*) may be defined by the large-lag asymptotic scaling of the auto covariance function [29]. For *H* ∈ (0.5, 1), *Y*(*t*) is said to be long-range temporally correlated (LRTC) whenever [29]:

$$\begin{array}{c} E\left(Y\left(t+s\right)Y\left(t\right)\right)-E{\left(Y(t)\right)}^{2}∼s^{2H-2} \end{array}$$ (8)

Informally, the larger *H*, the more *Y*(*t*) fluctuates over long time-scales. For auto covariances decaying faster than *s*^−1^, one defines *H* = 1/2 and *Y*(*t*) is not LRTC [29]; this means that over long time-scales, *Y*(*t*) may be treated as uncorrelated. From now on, we distinguish the Hurst exponents of *X*′(*t*) and *g*_*ω*_(*X*′(*t*)) as *H*_*raw*_ and $H_{amp}^{\omega }$. Thus *H*_*raw*_ is a measure of how much *X*′(*t*) fluctuates over long-time scales, whereas $H_{amp}^{\omega }$ is a measure of how much the amplitude envelopes of narrowbands of *X*′(*t*) fluctuate over long-time scales; note that there is no *a priori* reason that these fluctuations should be related—this is because a long-range correlated time-series (with high *H*) can be generated by filtering white noise whose narrow subbands have amplitudes with negligible autocorrelations) [30] but equally may be generated as the superposition of narrow subbands with long-range autocorrelations in their ampltiude envelopes.

Typically in neuroscientific applications Hurst exponents are measured with Detrended Fluctuation Analysis (DFA) [31]. Applying DFA allows us to quantify the fluctuations displayed in the bottom three panels of Fig 2. See Section: Materials and Methods: Detrended Fluctuation Analysis for a review of DFA.

The detrended cross correlation coefficient [32]*ρ*_*DCCA*_(*n*, *Y*_1_, *Y*_2_) is a measure of correlation between two time-series *Y*_1_(*t*) and *Y*_2_(*t*) at a time-scale *n*, which is invariant to non-stationary trends of a fixed polynomial degree. Informally *ρ*_*DCCA*_(*n*, *Y*_1_, *Y*_2_) measures to what degree fluctuations on the time-scale *n* co-occur between *Y*_1_ and *Y*_2_ and measures correlation but by ignoring non-stationary trends. When context leaves no room for ambiguity we abbreviate *ρ*_*DCCA*_(*n*, *Y*_1_, *Y*_2_) to *ρ*_*DCCA*_(*n*). Applying *ρ*_*DCCA*_(*n*) to pairs of amplitudes of narrowband activity *g*_*ω*_*i*__(*X*′(*t*)), *g*_*ω*_*j*__(*X*′(*t*)) allows us to quantify the correlations between the fluctuations in the amplitude envelopes displayed in the bottom two panels of Fig 2.

Given the measures of long-range variablity $H_{amp}^{\omega }$ and *ρ*_*DCCA*_(*n*) we are now ready to describe our theoretical results, which formalize the discussion of Section: Results: Overview of Theory.

**Fig 5:**
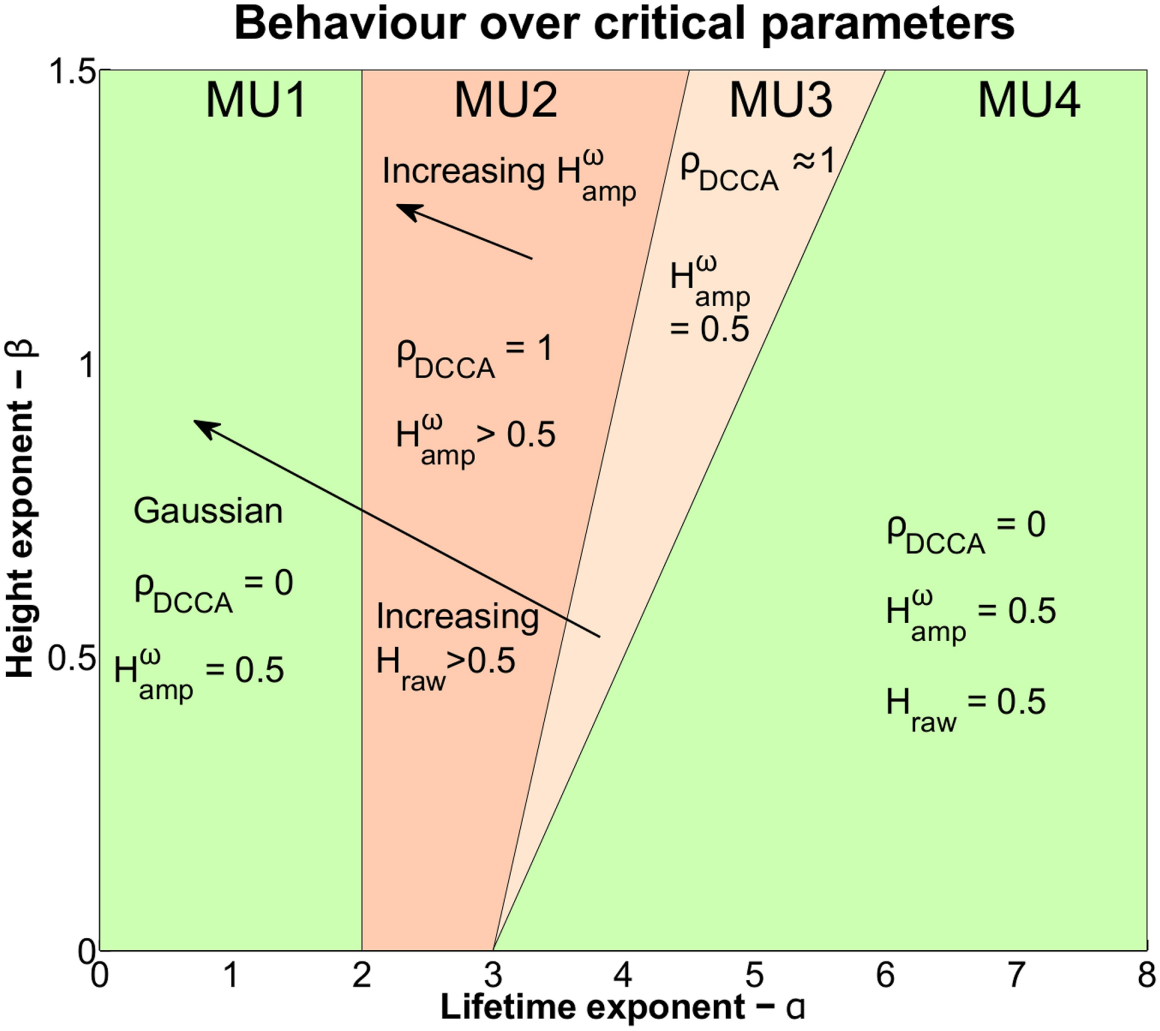
Division of critical exponents into meta-universality classes. The figure displays the range of qualitative behaviours we predict with our theory. Areas marked in green display no LRTC behaviour in sub-bands or DCCA correlations between sub-bands. Areas in red display LRTC and/or cross correlations between amplitudes of sub-bands ($H_{amp}^{\omega }=1/2$, *ρ*_*DCCA*_(*n*) = 0 for large *n*).

Our findings are summarized in Table 1 and Fig 5. Most importantly our theory makes rigorously quantified differential predictions between the PF and the CD theories. The differentiation between these models, and between groups of universality classes, which we term *meta-universality classes*, is made on the basis of the values of $H_{amp}^{\omega }$ and lim_*n*→∞_ *ρ*_*DCCA*_(*n*).

**Table 1:** Summary of our theoretical results.

model class	*α*	*β*	*H*_*raw*_	*H*_*amp*_	lim_*n*→∞_ *ρ*_*DCCA*_(*n*)
MU1	*α* ≤ 2	arbitrary	*β* − *α*/2 + 2	0.5	0
MU2	*α* > 2	>*α* − 3	*β* − *α*/2 + 2	*β*/2 − *α*/2 + 2	1
MU3	*α* > 2	≤*α* − 3 and $>\frac{\alpha -3}{2}$	*β* − *α*/2 + 2	0.5	1 (small *ω*_1_, *ω*_2_)
MU4	*α* > 2	$\leq \frac{\alpha -3}{2}$	0.5	0.5	0
PF	-	-	>0.5	0.5	0

For the PF theory we find that *H*_*amp*_ = 0.5 and lim_*n*→∞_ *ρ*_*DCCA*_(*n*) = 0. Thus according to PF, asymptotically, *g*_*ω*_(*X*′(*t*)) is not auto-correlated and *g*_*ω*_1__(*X*′(*t*)) and *g*_*ω*_2__(*X*′(*t*)) are uncorrelated over large time scales for distinct frequencies *ω*_1_ ≠ *ω*_2_.

For the CD theory we find that there are four meta-universality classes. In the first meta-universality class (MU1) which is defined by *α* ≤ 2 we find that *H*_*amp*_ = 0.5 and lim_*n*→∞_ *ρ*_*DCCA*_(*n*) = 0. On the other hand in the second meta-universality class (MU2), defined by *α* > 2 and *β* > *α* − 3, we find that $H_{amp}^{\omega }=\beta /2-\alpha /2+2$ and lim_*n*→∞_ *ρ*_*DCCA*_(*n*) = 1. In the third meta-universality class (MU3), defined by *α* > 2 and $\frac{\alpha -3}{2}<\beta \leq \alpha -3$, we find that *H*_*amp*_ = 0.5 and lim_*n*→∞_ *ρ*_*DCCA*_(*n*) → 1 as *n* → ∞. In the fourth meta-universality class (MU4), defined by *α* > 2 and $\beta \leq \frac{\alpha -3}{2}$, we find that *H*_*amp*_ = 0.5 and lim_*n*→∞_ *ρ*_*DCCA*_(*n*) = 0. Thus according to CD, for large values of *α* and small *β* we predict qualitatively identical long-range properties of *g*_*ω*_(*X*′(*t*)) as predicted by PF, but for intermediate values of *α* and large *β* we see non-trivial long-range auto-correlations and cross-correlations of *g*_*ω*_*i*__(*X*′(*t*)), *g*_*ω*_*j*__(*X*′(*t*)). For small values of *α* CD again predicts qualitatively identical long-range properties of *g*_*ω*_(*X*′(*t*)) as predicted by PF.

These results allow us to distinguish between the PF theory and CD theory on the basis of *H*_*amp*_ and *ρ*_*DCCA*_(*n*) in the classes MU2 and MU3.

### Simulations

For the CD model, we model the activity of local networks by:

$$\begin{array}{c} a(t)=b(t)+c(t)\epsilon (t) \end{array}$$ (9)

*b*(*t*) is the average avalanche shape, *c*(*t*) the shape of the variance profile and *ϵ*(*t*) a coloured noise with the spectrum known for a critical system $P\left[\omega \right]∼\omega^{-\beta -1}$ [3].

We check the predictions of the PF model, by modelling the observed activity *X*′(*t*) as filtered uncorrelated white noise, to yield a process with a power-law power-spectrum; this is because with exponentially decaying avalanche height and size distributions, as claimed by the PF theory, long-range properties are identical to those of white noise. For details see Section: Materials and Methods Simulation Details.

Software for our analyses is available in S1 Software.

#### Examples

**Fig 6:**
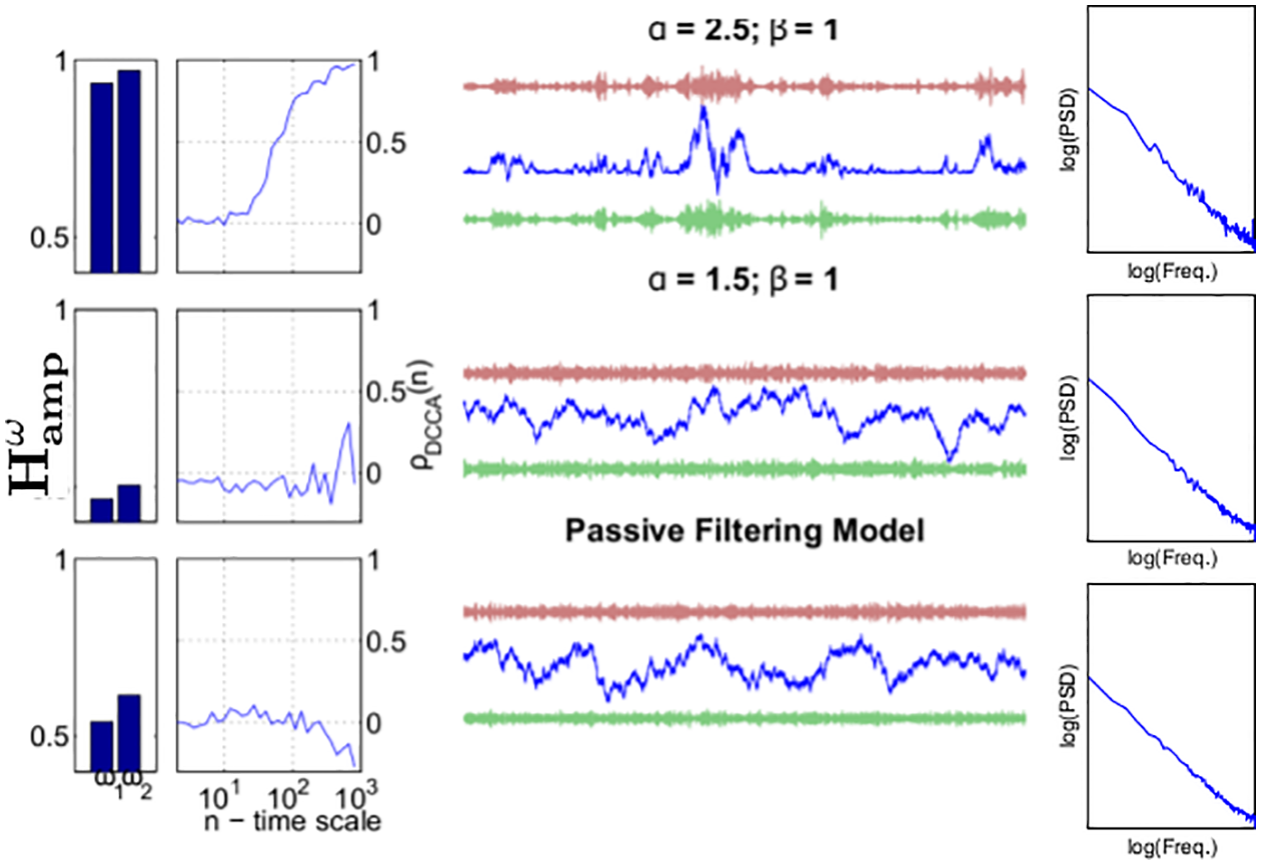
Examples illustrating our theory. Centre: Sample paths from the PF and CD models. In each of the three cases the *x*-axis denotes time and the *y*-axis number of activations. For each panel the middle trace denotes *X*′(*t*) and the top and bottom *g*_*ω*_*i*__(*X*′(*t*)). Left: DCCA correlation coefficients *ρ*_*DCCA*_(*n*) and Hurst exponents $H_{amp}^{\omega }$. Right: Power-spectra of *X*′(*t*) in each of the three cases. We see that the top row displays qualitatively different properties to the middle and bottom rows, although the power-spectra are the same in each case. The differences are, however, well quantified by the Hurst exponent and *ρ*_*DCCA*_(*n*) in the left-hand column. See Section: Results: Simulations: Examples.

In Fig 6 we first present three examples illustrating our theoretical predictions from the PF model and the CD model in MU1 and MU2. Bottom row: we sample from the PF model with *γ* = 1.9, and *T* = 40000 (right column). We measure DCCA correlations (middle column) between amplitudes in the frequency ranges [0.68, 0.72] and [0.78, 0.82] of half the sampling frequency (forwards and backwards filtering with Butterworth filters of order 2). with *n* log-spaced between 20 and 9000 and Hurst exponents $H_{amp}^{\omega }$ (left column) of the same amplitudes using DFA with window sizes between 1000 and 9000. We confirm that to within inaccuracies generated by the finite sample size, $H_{amp}^{\omega }=0.5$ and *ρ*_*DCCA*_(*n*) → 0 as *n* → ∞. Top two rows: we generate two processes assuming CD according to Eq (1) generating avalanches as described above. We set *q* = 5, *T* = 40000, *β* = 1 and *α* = 2.5, 1.5 (top, middle resp.). We confirm that for *α* = 2.5, *H*_*amp*_ > 0.5 and *ρ*_*DCCA*_(*n*) → 1 as *n* → ∞. On the other hand, for *α* = 1.5 we confirm that to within inaccuracies generated by the finite sample size, *H*_*amp*_ = 0.5 and *ρ*_*DCCA*_(*n*) → 0 as *n* → ∞. These quantitative differences in *g*_*ω*_(*X*′(*t*)) between the three conditions is reflected in the qualitative nature of the data. Whereas for the PF model and CD model with *α* = 1.5, the data take the form of a homogeneous random walk, for the CD model with *α* = 2.5, the structure of the time-series is more heterogeneous, with large avalanches visible among the many avalanches triggered.

#### PF model

The aim of this simulation is to verify that the behaviour confirmed in the preceding example for the PF model is reproducible, satisfying $H_{amp}^{\omega }=0.5$ and lim_*n*→_ *ρ*_*DCCA*_(*n*) = 0. We simulate *X*′(*t*) from the PF model with *T* = 15000, 100 times. In each case the data are then filtered forward and backwards in two separate frequency bands (between 0.39 and 0.41 of the sampling frequency and 0.29 and 0.31 of the sampling frequency) with Butterworth filters of order *m*. We then measure DCCA correlations between the Hilbert transforms *g*_*ω*_(*X*′(*t*)) of these signals and measure their Hurst exponents $H_{amp}^{\omega }$ with DFA, in both cases using window lengths between 10^3^ and 10^4^. We repeat this setup for *γ* = 0.8, 1.8 and *m* = 2, 4.

**Fig 7:**
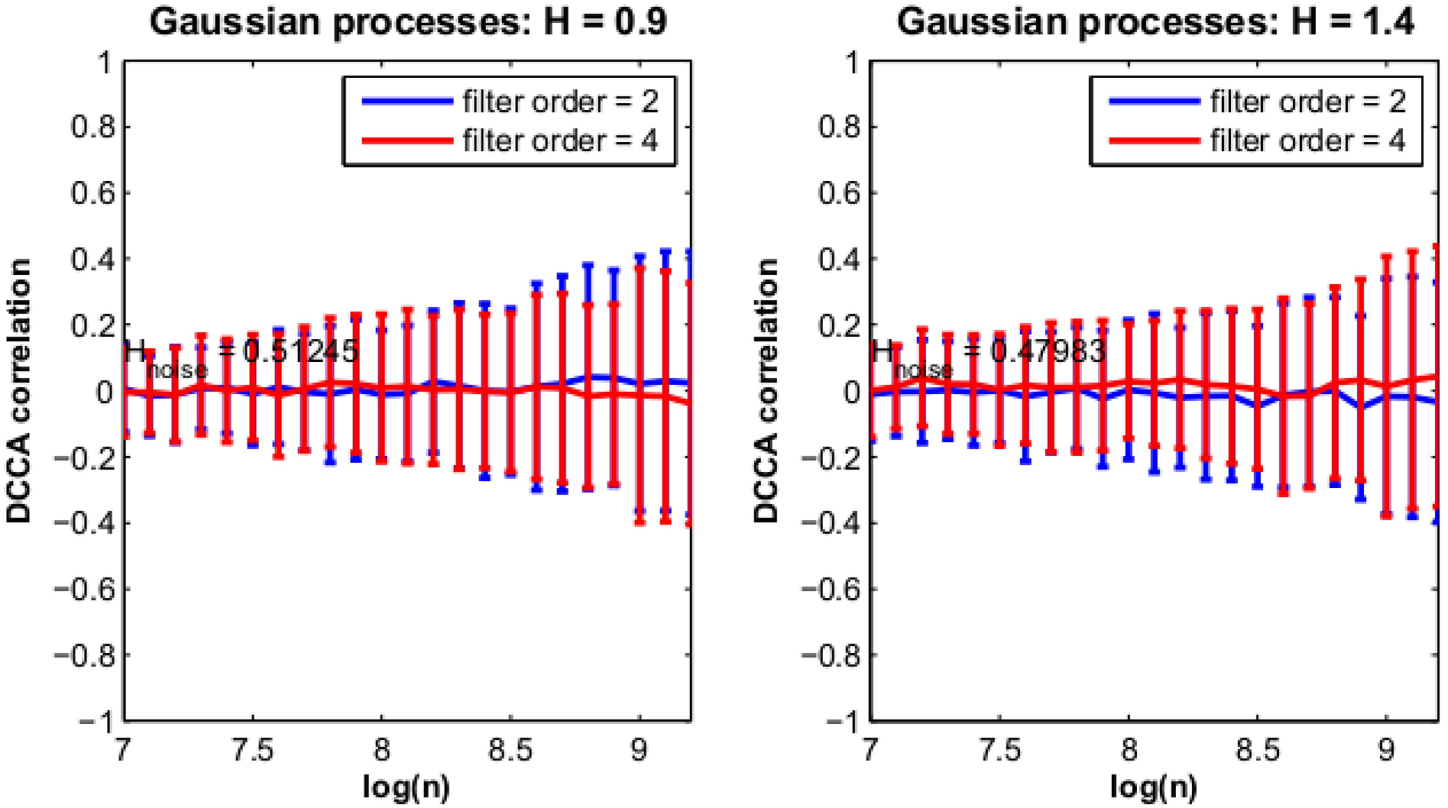
Simulation for PF model. The figure displays the results averaged over 100 iterations for the PF model in simulation. The left hand panel and the right hand panel correspond to differing values of Hurst exponent of the process sampled. In each case $H_{amp}^{\omega }$ and *ρ*_*DCCA*_(*n*) are estimated and averaged. The trace corresponds to *ρ*_*DCCA*_(*n*) whereas $H_{amp}^{\omega }$ is displayed in text. See Section: Results: Simulations: PF model.

The results are displayed in Fig 7 and show that, although there are small sample effects, on average we obtain *H*_*amp*_ = 0.5 and zero cross correlations *ρ*_*DCCA*_(*n*) = 0 between frequency bands.

#### Predictions for all meta-universality classes

**Fig 8:**
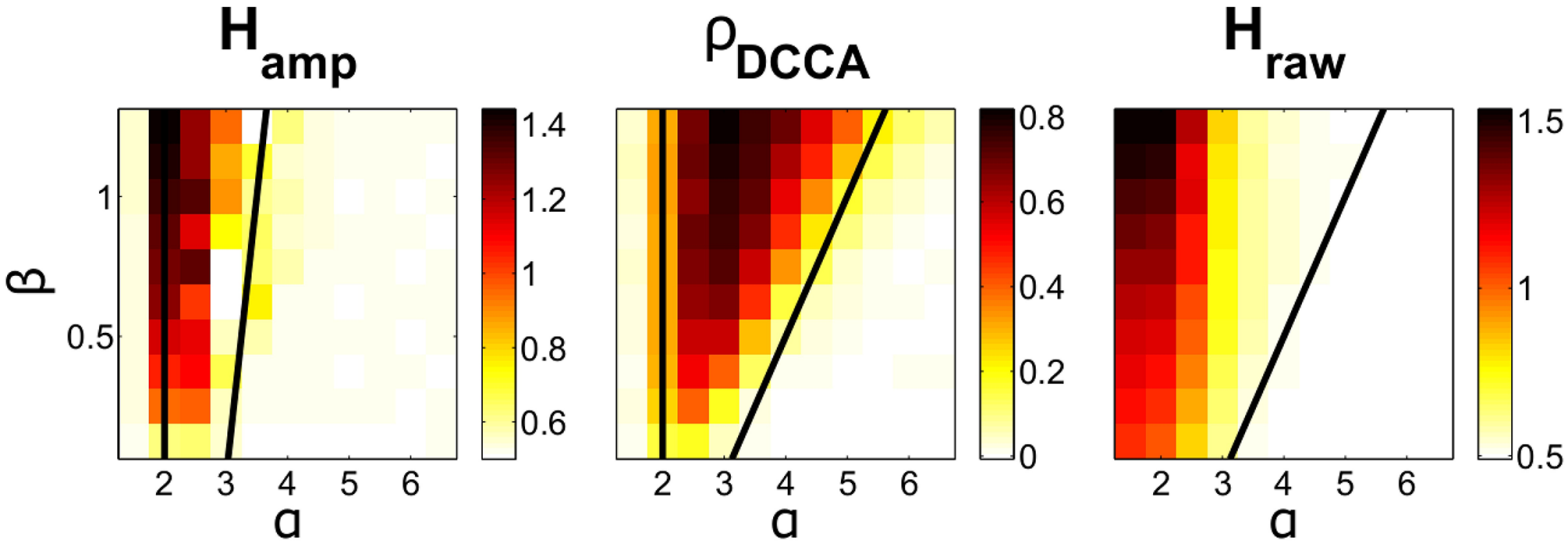
Simulation for CD model over universality classes. DCCA correlation coefficients and Hurst exponent for the simulated CD model. The *x*-axis denotes the exponent *α* and the *y*-axis denotes *β*. The black lines denotes the transitions in meta-universality class according to the theory. In each case the colour on the image and colourbar corresponds to the quantity in the subtitle (e.g. in the left hand image the colour corresponds to $H_{amp}^{\omega }$.) The details of the simulation are given in Section: Results: Predictions for all Meta-Universality Classes.

The aim of this simulation is to confirm the quantitative theoretical predictions for CD displayed in Table 1 across universality classes. For each pair of exponents in the ranges *α* = 1.5, 2.0, …, 6.5 and *β* = 0.25, 0.5, …, 3, we generate a sample path *X*′(*t*) of length *T* = 300,000, with a cutoff at *L*_*c*_ = 100,000, and number of superpositions *q* = 5. The first 100,000 time points are discarded, to ensure stationarity. We then design Butterworth filters of order 2 between 0.29 and 0.31 (*ω*_1_ = 0.3) and between 0.39 and 0.41 (*ω*_2_ = 0.3) of the sampling frequency. The data from *X*′(*t*) are then filtered forwards and backwards and the amplitude envelopes are calculated *g*_*ω*_1__(*X*′(*t*)) and *g*_*ω*_2__(*X*′(*t*)) (yielding effective filter order 4). We measure the Hurst exponent of *g*_*ω*_1__(*X*′(*t*)), using DFA, and the DCCA correlation coefficients between *g*_*ω*_1__(*X*′(*t*)) and *g*_*ω*_2__(*X*′(*t*)) setting *n* to log spaced values between 100 and 200000. This setup is repeated 100 times, and the results of the simulations are averaged. The results of this simulation for all critical parameters are displayed in Fig 8. We find good agreement between the theory for each meta-universality classes and the simulation results.

#### Further simulations

“Further Simulations” of S1 Appendix provides details of two further sets of simulations. The first verifies the accuracy of the theoretical exponent relations described in Table tab:theory (“Exponent Relations” in S1 Appendix). The second investigates the effect of signal-to-noise ratio on accuracy of the theoretical relations derived for data contaminated by noise (“The Effect of Signal-to-Noise Ratio” in S1 Appendix).

### Analysis of EEG recordings of the human brain

Finally we tested the PF and CD hypotheses by estimating $H_{amp}^{\omega }$, *H*_*raw*_ and *ρ*_*DCCA*_(*n*) on the experimental EEG data of seven human subjects. Section: Materials and Methods: Experiment describes the Experiment and our data preprocessing protocols in detail.

**Fig 9:**
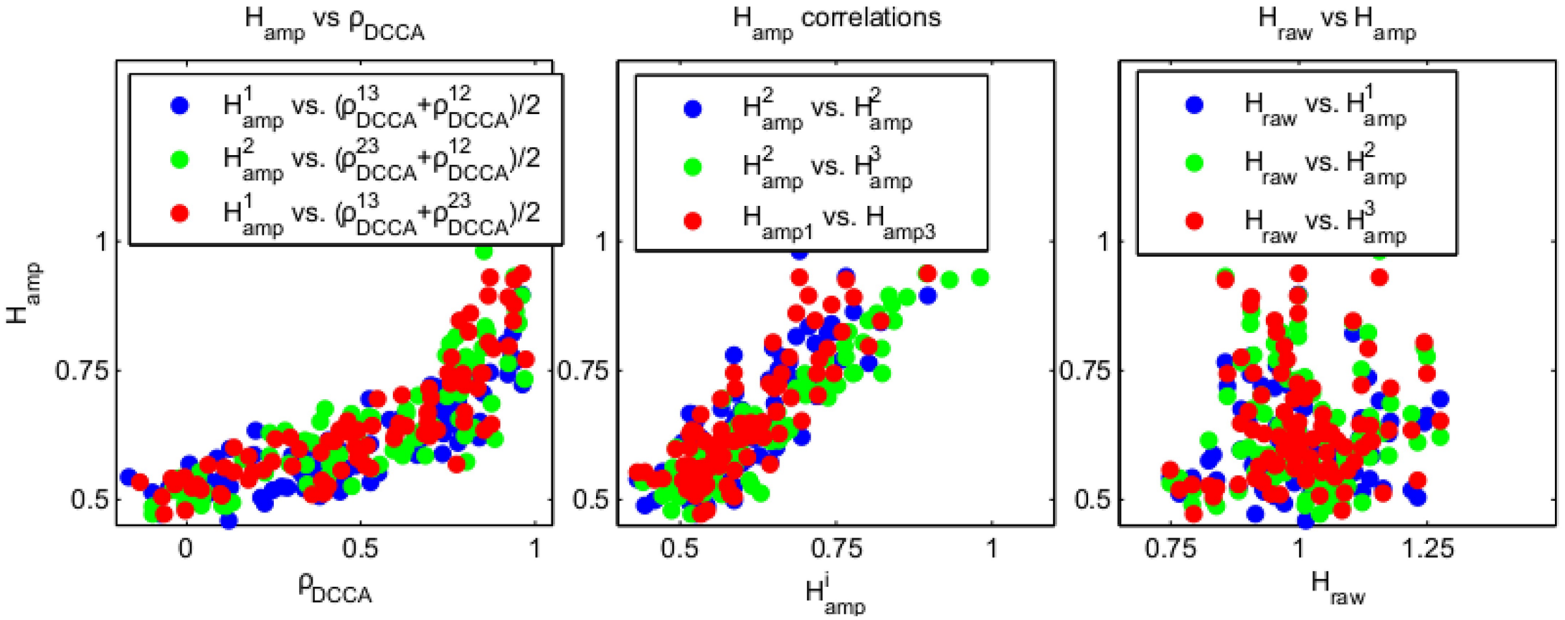
Results of data analysis of human EEG. The frequency ranges analysed *i* = 1, 2, 3 are 35-40Hz, 60-65Hz and 72-77 Hz respectively, which are displayed as superscripts in the plots. Each point on a plot corresponds to estimates made from one EEG spatially filtered component (SSD), with the colours denoted distinct frequencies. In the left hand panel we display *ρ*_*DCCA*_(*n*) values at the highest scale vs. $H_{amp}^{\omega }$ values. In the middle panel we display $H_{amp}^{\omega }$ values between frequencies and in the right hand panel we display $H_{amp}^{\omega }$ plotted vs. *H*_*raw*_.

The results of our analysis over all subjects are displayed in Fig 9. For 244 of 261 signals, the DFA estimate of $H_{amp}^{\omega }$ is higher than 1/2, clearly indicating the presence of LRTC (median $H_{amp}^{\omega }$ = 0.61, 5th and 95th percentiles 0.49 and 0.85, p < 0.0001). Likewise, 238 of 261 DCCA correlation coefficients *ρ*_*DCCA*_(*n*) at the highest scale *n* between frequency ranges were positive (*p* ≪ 0.0001). We measured the gradient of *ρ*_*DCCA*_(*n*) against log(*n*) for the largest 11 time-scales considered. We found that in 82% of cases, the gradient is positive, indicating that if the range of *n* considered were extended in the presence of more data, the *ρ*_*DCCA*_(*n*) values would continue to approach 1. We also found that a large proportion of *ρ*_*DCCA*_(*n*) values are larger than 0.9. These results provide strong evidence in support of the theoretical prediction for CD, lim_*n*→∞_ *ρ*_*DCCA*_(*n*) = 1. We found moreover that the *ρ*_*DCCA*_ values at the highest scale and $H_{amp}^{\omega }$ measured from the same neural data were highly correlated (*p* ≪ 0.0001) and $H_{amp}^{\omega }$ values in distinct frequency ranges were highly correlated (*p* ≪ 0.0001) (likewise for *ρ*_*DCCA*_(*n*)). We also found that the *ρ*_*DCCA*_(*n*) and $H_{amp}^{\omega }$ values were not significantly correlated with *H*_*raw*_ (*p* > 0.05). Finally we measured the correlation between the average *ρ*_*DCCA*_(*n*) value over frequencies for a component versus a surrogate measure for the signal noise to ratio, namely the ratio of power in the alpha range 8–13Hz to power in a wider frequency range 5–16Hz. This measure determines to what extent the alpha rhythm stands above the 1/*f*^*γ*^ shape of the power-spectrum (see Fig F in S1 Appendix). We found that the average *ρ*_*DCCA*_(*n*) value was positively correlated with the signal-to-noise measure (Spearman correlation: *r* = 0.32, *p* = 0.0026).

These results favour the CD hypothesis over the PF hypothesis. This is because, according to the PF hypothesis, $H_{amp}^{\omega }=0$ and lim_*n*→∞_ *ρ*_*DCCA*_(*n*) = 0. On the other hand, in MU2, *H*_*amp*_ > 0.5 and lim_*n*→∞_ *ρ*_*DCCA*_(*n*) = 1. and in MU3, lim_*n*→∞_ *ρ*_*DCCA*_(*n*) → 1 for low frequencies. It might be argued that these results are explained by the PF hypothesis, since *ρ*_*DCCA*_(*n*) → 0 is only true asymptotically, and for finite *n* we could have *ρ*_*DCCA*_(*n*)>0. However, this is not a sufficient explanation, since we also find empirically that $H_{amp}^{\omega }>1/2$ as is only possible in MU2, with many values far larger than 1/2; in addition, we find that the gradient of the graphs of *ρ*_*DCCA*_(*n*) against log(*n*) are positive in over 80% of cases and in many cases the values of *ρ*_*DCCA*_(*n*) are indeed close to 1. Thus the data are better explained by CD in terms of the finiteness of *n*, with the gradient indicative of the limit *ρ*_*DCCA*_(*n*) → 1 rather than *ρ*_*DCCA*_(*n*) → 0 (see the middle column of Fig 6 for an illustration). Moreover, in MU3, we *do not* expect that *ρ*_*DCCA*_(*n*) is close to one, but rather greater than 0, in the finite sample (see Fig 8); the theory asserts that this is only true for the low-frequency, large scale limit.

Another respect in which these data confirm the CD hypothesis is the fact that the theory predicts the same exponent for each frequency band $H_{amp}^{\omega }=\alpha +\beta /2+2$, not merely that $H_{amp}^{\omega }>1/2$, and also that high *ρ*_*DCCA*_(*n*) values and $H_{amp}^{\omega }$ values coincide. The high correlation between frequencies between the $H_{amp}^{\omega }$ values (middle panel of Fig 9) and the correlation between $H_{amp}^{\omega }$ values and *ρ*_*DCCA*_(*n*) values (left-hand panel of Fig 9) thus lend extra-evidence to the hypothesis of CD.

#### Further data analysis

In “Further Data Analysis” in S1 Appendix we investigate the impact of the spatial filters chosen, confirming that the choice made is not critical, and describe computations of power-spectra for each of the subjects, providing additional insight into the dataset, and provide full DFA plots for one of the subjects analysed, demonstrating the scale-free nature of the data analysed.

## Conclusion

Our findings provide evidence which support the hypothesis of criticality in the human brain rather than the shaping of a 1/*f*^*γ*^ spectrum by passive filtering; passive filtering of neural signals may generate a power-law spectrum but will not induce LRTC of narrowband amplitudes or DCCA correlations between narrowband amplitudes.

Such conclusions are made possible by our theory of meta-universality classes. Depending on the values of critical exponents, we predict qualitatively differing behaviours, which fall into four classes. These behaviours are manifest in the amplitude envelopes of narrowband processes of the measured data.

In contrast to the methods applied in previous studies on criticality in neuroscience, our framework does not depend on being able to detect single avalanches. Indeed our theory shows that if the network falls into the first meta-universality class (MU1), then single avalanches cannot be discernible in principle. Thus, if no avalanche dynamics are detected from macroscopic recordings, then this does not mean the system is not critical. Thus *it is a fallacious argument to claim that no avalanches are discernible from macroscopic recordings means there must be another explanation for 1/f power-spectra.* This fact may explain the failure to detect avalanche dynamics in the experiments of [10, 17].

Several papers have attempted to circumvent the problems due to the lack of separation of time-scales by thresholding time-series obtained with macroscopic neural recordings. On the basis of our analysis we advise here that caution should be exercised when using such thresholding approaches. If the network is critical close to the transition from MU2 to MU1 then the estimates of the critical exponents *α* and *β* obtained in this way are potentially biased since individual avalanches are not discernible. In particular if *α* < 2 then such analysis will yield an estimate that no critical dynamics are present at all although they are present but not identifiable. Moreover continuity considerations may imply that for *α* close to 2 estimation could be inaccurate.

Given these difficulties relating to continuous neural data what do existing analyses which use thresholding tell us? Several papers have confirmed predictions of the CD hypothesis over short time-scales and for the extremal activity of macroscopic neuronal time-series such as EEG/ MEG [13–15]. Moreover exponents correlate with Hurst exponents of e.g. alpha range amplitude envelopes [15]. Our findings imply that close to the boundary MU1/2, avalanches are impossible to detect individually; however close to the border between MU2/3 avalanches should nevertheless be detectable at the extremes of neuronal time-series, for sufficiently large *β*, assuming artifactual activity leading to shifts in voltage is not present. However, since avalanches in EEG/MEG are estimated from broad-band data, it is still unclear what effect passive filtering will have on the derived exponent values—this awaits further work, thus leading us to question the size exponent *τ* = −3/2 reported in several studies. In addition, even if this value is accurate, the theory which claims *τ* = −3/2 is only valid for a critical branching process or mean-field network [33]; it is not entirely clear whether the brain should be modelled as such. Although Hurst exponents in the alpha-range are seen to correlate with estimated avalanche exponents [15], the mechanism responsible for this correlation is unclear, as these oscillations occur at a fixed frequency and criticality is a scale free phenomena. Finally, our work shows that if the thresholding technique fails to detect avalanche dynamics as reported by [17], this does not mean that criticality is not present—our results present a potentially more illuminating test capable of testing scaling over several orders of magnitude on a sound theoretical basis, although in MU1 our methods will also fail to detect criticality if present.

Another interesting question is, can we in principle derive the exact values of the exponents *α* and *β* from the data, without measuring avalanche dynamics but by considering only the Hurst exponents $H_{amp}^{\omega }$ and *H*_*raw*_? The answer is, in principle affirmative in MU2, but not otherwise. In MU2 we have bijective relations between $H_{raw},H_{amp}^{\omega }$ and *α*, *β*, whereas in the other meta-universality classes we have $H_{amp}^{\omega }=0.5$. Thus in MU2 in principle we can estimate *α* and *β* by measuring *H*_*raw*_ and *H*_*amp*_ and inverting the theoretical relations to *α* and *β* which we derived (see Table 1). However, in practice this is problematic, since signal to noise ratio distorts the empirically measured exponents towards 0.5 [34]. This does not affect the qualitative conclusion, that if $H_{amp}^{\omega }>0.5$ then we must have a network in MU2, but it skews the ability to exactly estimate *α* and *β*.

Nonetheless, using our framework and despite the problems of signal-to-noise ratio, we are able differentiate between the meta-universality classes, thus providing a range of confidence on the possible universality class—CD predicts that only MU2 has $H_{amp}^{\omega }>0.5$. On this basis we find empirically at least some brain regions must be in MU2. On the other hand at least some values of *ρ*_*DCCA*_(*n*) and $H_{amp}^{\omega }$ measured are not consistent with MU2. [5] find a value of *α* = 1.7±0.2, which means the network lies in MU1—in combination with our own results, this provides evidence that a range of critical exponents may be relevant to understanding brain function.

## Materials and methods

### Amplitudes estimated with the Hilbert transform

Let *f*_*ω*_(⋅) be a linear narrowband filter with pass band [*ω* − Δ, *ω* + Δ], and H(·) the Hilbert transform then:

$$\begin{array}{c} g_{\omega }\left(X\left(t\right)\right)=\left|f_{\omega }\left(X\left(t\right)\right)+iH\left(f_{\omega }\left(X\left(t\right)\right)\right)\right| \end{array}$$ (10)

When we want to make the pass band explicit we write *f*_*ω*,Δ_(*X*(*t*)) and *g*_*ω*,Δ_(*X*(*t*)). When we estimate *g*_*ω*_(*X*(*t*)) in software, we use the MATLAB code abs(hilbert(⋅)).

### Detrended fluctuation analysis

Detrended Fluctuation Analysis (DFA) [31] is a methodology for the estimation of the Hurst exponent *H* of a (possibly non-stationary) time-series. Its advantage over covariance analysis or analysis of the power-spectrum are its robustness to trends contaminating the empirical time-series and its desirable convergence properties [35].

The steps involved in DFA are as follows. First one forms the aggregate sum of the empirical time-series *X*(*t*):

$$\begin{array}{c} x\left(t\right)=\sum_{i=1}^{t}X\left(i\right) \end{array}$$ (11)

(From now on whenever we use lower case for a time-series, e.g. *x*(*t*), we mean the time-series obtained from the corresponding upper case time-series, e.g. *X*(*t*), by way of this operation.) Analysis of the fluctuations in *X*(*t*) may then be performed by measuring the variance of *x*(*t*) in windows of varying size *n* *after* detrending, i.e., *x*(*t*) is split into windows of length *n*, $x_{n}^{\left(1\right)},\cdots ,x_{n}^{\left(j\right)},\cdots ,x_{n}^{\left(\lfloor N/n\rfloor \right)}$ and the average variance after detrending the data of *x*(*t*) in these windows is formed; i.e. let *P*_*d*_ be the operator which performs least squares detrending of polynomial degree *d*, then the DFA coefficients or detrended variances of degree *d* are:

$$\begin{array}{c} F_{DFA}^{2}\left(n\right)= \end{array}$$ (12)

$$\begin{array}{c} \frac{1}{\lfloor N/n\rfloor \cdot n}\sum_{j}{\left(x_{n}^{\left(j\right)}-P_{d}\left(x_{n}^{\left(j\right)}\right)\right)}^{⊤}\left(x_{n}^{\left(j\right)}-P_{d}\left(x_{n}^{\left(j\right)}\right)\right) \end{array}$$ (13)

**Fig 10:**
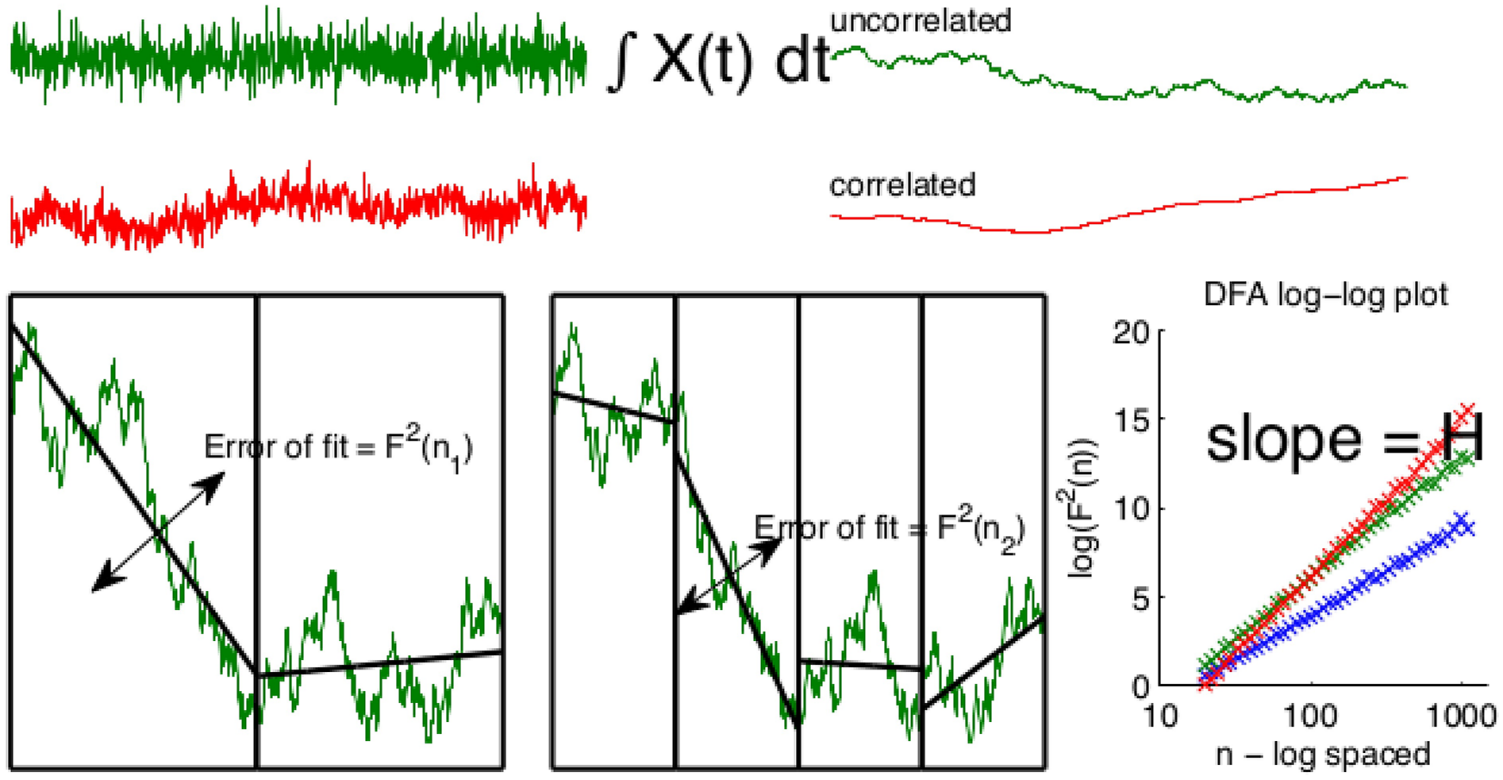
Illustration of DFA. Top left: two signals *X*_*i*_(*t*) uncorrelated (green) and LRTC (red) from top to bottom with Hurst exponents *H*_1_ = 0.5 < *H*_2_. Top-right: the cumulative sum *x*(*t*) of the original signal, which display differing random walk behaviours. Bottom left: the DFA coefficients $F_{DFA}^{2}\left(n\right)$ are estimated by detrending *x*(*t*) in time windows of length *n* and estimated the error of the linear fit. Botton right *F*^2^(*n*) *n*^2*H*^ allowing *H* to be estimated by regression in log coordinates.

Crucially, in the limit of data the slope of $\text{log}(F_{DFA}^{2}\left(n\right))$ against log(*n*) converges to *H*. Thus *X*(*t*) is LRTC if and only if the estimate of *H*, $\hat{H}$, converges to a number greater than 0.5 in the limit of data. We note here that there are numerous methods for the estimation of the Hurst exponent; these include wavelet estimators [35, 36], log-periodogram based methods [37], among others [38]. We use DFA since it is standard in the physics and neuroimaging literature, and yields competitive estimates [39]. See Fig 10 for an illustration of DFA. [40] contains a useful review and explanation of DFA.

### Detrended cross-correlation analysis

Podobnik and Stanley propose Detrended Cross-Correlation Analysis (DCCA) [41], an extension of DFA to two time-series, by considering the detrended covariance:

$$\begin{array}{c} F_{DCCA}^{2}\left(n\right)= \end{array}$$ (14)

$$\begin{array}{c} \frac{1}{\lfloor N/n\rfloor \cdot n}\sum_{j}{\left({\left(x_{1}\right)}_{n}^{\left(j\right)}-P_{d}\left({\left(x_{1}\right)}_{n}^{\left(j\right)}\right)\right)}^{⊤}\left({\left(x_{2}\right)}_{n}^{\left(j\right)}-P_{d}\left({\left(x_{2}\right)}_{n}^{\left(j\right)}\right)\right) \end{array}$$ (15)

DCCA quantifies the behaviour of the covariance between *X*_1_ and *X*_2_ over a range of time-scales given by *n* and generalizes DFA in the sense that if *X*_1_ = *X*_2_ then $F_{DCCA}^{2}\left(n\right)=F_{DFA}^{2}\left(n\right)$.

In analogy to the Pearson correlation coefficient, the *detrended cross correlation coeffcient* is [32]:

$$\begin{array}{c} \rho_{DCCA}\left(n,X_{1},X_{2}\right)=\frac{F_{DCCA}^{2}\left(n\right)}{\sqrt{F_{DFA}^{2}{\left(n\right)}_{X_{1}}}\sqrt{F_{DFA}^{2}{\left(n\right)}_{X_{2}}}} \end{array}$$ (16)

i

**Fig 11:**
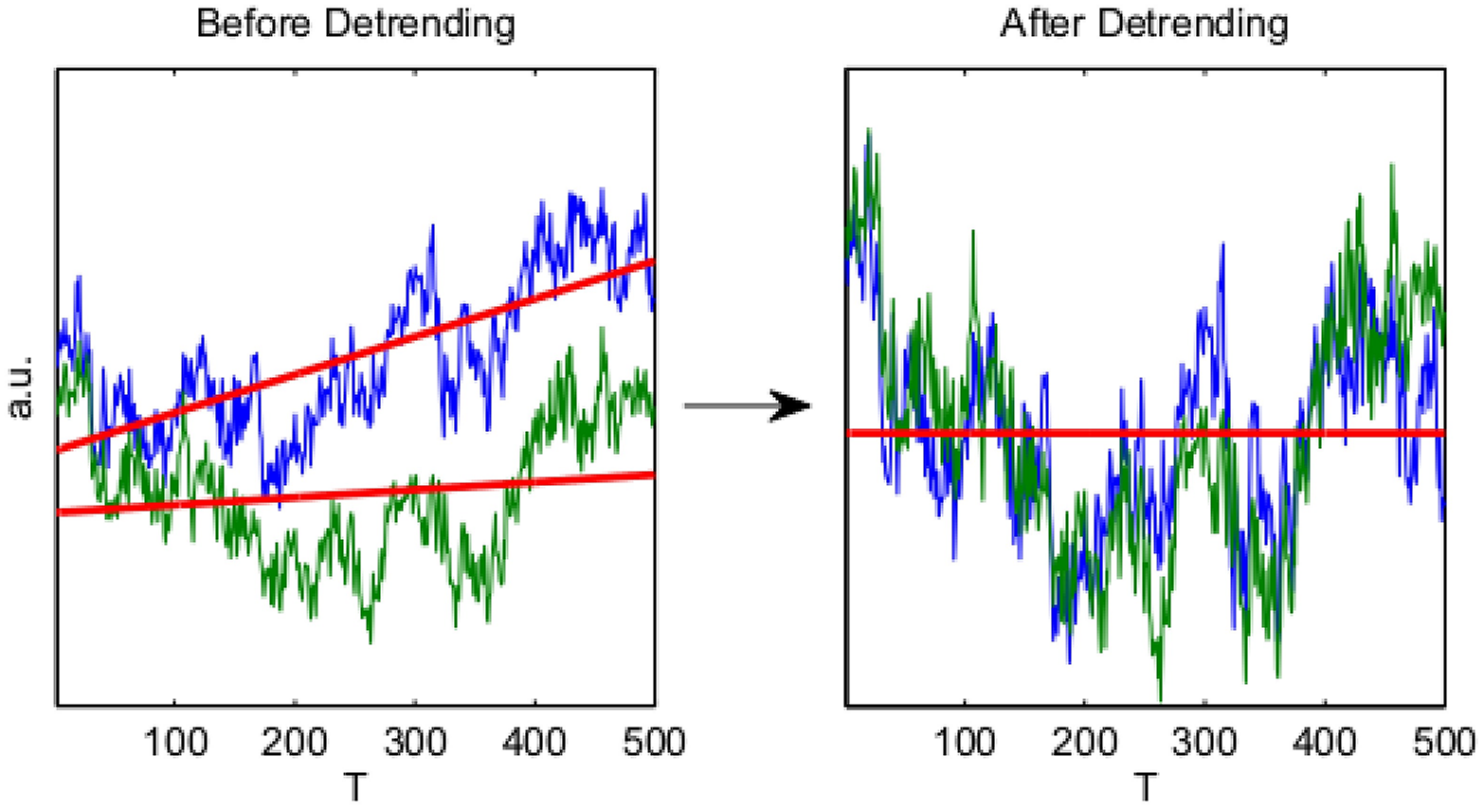
Illustration of DCCA. DCCA is the exact analogy of DFA (see Fig 10) for two time-series. The left hand panel displays the cumulative sum of two time-series; the right hand panel displays the same time-series after detrending. The strong correlation between the time-series only becomes apparent after detrending, explaining the advantage of DCCA for long-range dependent and non-stationary time-series.

*ρ*_*DCCA*_ quantifies the correlation between *X*_1_ and *X*_2_ over a range of time-scales. Note that while estimation of *ρ*_*DCCA*_ is technically more complex than for Pearson correlation, v both coefficients estimate the *same* quantity for stationary time-series, not contaminated by trends [42]. Thus *ρ*_*DCCA*_ generalizes the Pearson correlation coefficient. Applicability to non-stationary time-series is particularly important for the neural data analysis. Whenever the context allows for no ambiguity we abbreviate *ρ*_*DCCA*_(*n*, *X*_1_, *X*_2_) to *ρ*_*DCCA*_(*n*). An illustration of DCCA is provided in Fig 11.

### Details for Fig 3

We describe here how to reproduce Fig 3. We sample 5 × 6000 = 30000 instances of *a*(*t*) with unit length and unit expected height, with power-spectrum $P\left[\omega \right]∼\omega^{-\beta -1}$. Subsequently for *α* = 2, 2.125, 2.25, …, 5.5, at each time point *q* = 5 avalanches are initiated according to the model; however, for each universality class considered, the *a*_*i*,*s*_(*t* − *s*) are identical, taken from the 30000 pre-generated *a*(*t*), differing only in their length and height.

### Theory

#### Predictions for the PF theory

The results in this section are derived here for the first time unless otherwise stated/ cited.

Our first result is that for the *X*′(*t*) of the PF hypothesis $H_{amp}^{\omega }=0.5$ and, when *ω*_1_ ≠ *ω*_2_, *ρ*_*DCCA*_(*n*, *g*_*ω*_1__(*X*′(*t*)), *g*(*ω*_2_)(*X*′(*t*))) → 0 as *n* → ∞. This implies that *g*_*ω*_(*X*′(*t*)) and *g*_*ω*_(*X*(*t*′)) are uncorrelated for large *t* − *t*′ and that for distinct *ω*_1_ ≠ *ω*_2_ the narrowband amplitudes *g*_*ω*_1__(*X*′(*t*)) and *g*_*ω*_2__(*X*′(*t*)) are unrelated (left, bottom two rows of Fig 2).

This can be seen by splitting Eq (1) into avalanches which last longer than some value *L*′ and avalanches which have duration shorter than or equal to *L*′:

$$\begin{array}{c} X\left(t\right)=\sum_{L_{s,i}\leq L^{\prime }}h_{s,i}a_{s,i}\left(\frac{t-s}{L_{s,i}}\right)+\sum_{L_{s,i}>L^{\prime }}h_{s,i}a_{s,i}\left(\frac{t-s}{L_{s,i}}\right) \end{array}$$ (17)

The variance of the left hand term dominates because, if *M* is the number of avalanches active:

$$\begin{array}{ccc} \text{var}[\sum_{L_{s,i}\leq L^{\prime }}h_{s,i}a_{s,i}\left(\frac{t-s}{L_{s,i}}\right)] & ∼ & \#\left\{L_{s,i}\leq L^{\prime }\right\}\;\text{var}\left(h\right)\\ & ∼ & \#\{L_{s,i}\leq L^{\prime }\}\;C\\ & ∼ & \text{Pr}(L_{s,i}\leq L^{\prime }\}\;C\;M\\ & ∼ & C\;M \end{array}$$ (18)

Whereas:

$$\begin{array}{ccc} \text{var}[\sum_{L_{s,i}\geq L^{\prime }}h_{s,i}a_{s,i}\left(\frac{t-s}{L_{s,i}}\right)] & ∼ & \text{Pr}(L_{s,i}>L^{\prime }\}\;C\;M\\ & ∼ & \exp(-AL^{\prime })\;C\;M \end{array}$$ (19)

Therefore:

$$\begin{array}{c} f_{\omega }\left(X\left(t\right)\right)∼\sum_{L_{s,i}\leq L^{\prime }}h_{s,i}f_{\omega }\left(a_{s,i}\left(\frac{t-s}{L_{s,i}}\right)\right) \end{array}$$ (20)

Since time-points in this expression spaced more than *L*′ points apart are independent we also have that time-points of *g*_*ω*_(*X*(*t*)) spaced more than *L*′ points apart are independent, so that amplitude envelopes are asymptotically not autocorrelated, $H_{amp}^{\omega }=1/2$.

Moreover *ρ*_*DCCA*_(*n*, *g*_*ω*_1__(*X*(*t*)), *g*_*ω*_2__(*X*(*t*))) → 0 as *T* → ∞ since the numerator of the DCCA correlation coefficient is given by the DCCA coefficients $F_{X_{1},X_{2}}^{2}\left(n\right)$ and the denominator by $\sqrt{F_{X_{1},X_{1}}^{2}\left(n\right)F_{X_{2},X_{2}}^{2}\left(n\right)}$. The numerator behaves like the coefficients of white noise for large window sizes [43] and therefore tends to 0 in the limit. The terms in the denominator tend to a non-zero limit [44], so the entire quotient tends to 0. Since the effect of the filter of Eq (4) is simply to reweight *f*_*ω*_(*X*(*t*)) [23], then the result carries over to *g*_*ω*_(*X*′(*t*)).

#### Predictions for the CD theory

We assume that all power-law distributions are cut off at a lifetime *L*_*c*_ which is proportional to the size of the network considered and, for simplicity, that a fixed number *q*_*s*_ = *q* of avalanches begin at each time-point.

(**MU1**) *α* < 2

For *α* < 2 the number of avalanches active at time *t* is:

$$\begin{array}{ccc} & & \sum_{s=0}^{L_{c}}\#\left\{L_{t-s,i}|L_{t-s,i}>s\right\} \end{array}$$ (21)

$$\begin{array}{ccc} & & \;∼\int_{0}^{L_{c}}q\left(\int_{t^{\prime }}^{L_{c}}L^{-\alpha }dL\right)dt^{\prime }\\ & & \;∼\left(1-\alpha \right){\left(L_{c}\right)}^{1-\alpha }-\left(1-\alpha \right)\int_{0}^{L_{c}}{\left(t^{\prime }\right)}^{1-\alpha }dt^{\prime }\\ & & \;=\left(1-\alpha \right){\left(L_{c}\right)}^{1-\alpha }-\left(1-\alpha \right)\left(2-\alpha \right)\left(L_{c}^{2-\alpha }-1\right)\\ & & \;∼L_{c}^{2-\alpha } \end{array}$$ (22)

The first line follows by definition and the second line is an asymptotic approximation of the first line for large *L*_*c*_. Hence the number of avalanches active, $a_{s,i}\left(\frac{t-s}{L_{s,i}}\right)>0$, at any given time is unbounded in the system size. I.e., for times *s*_1_, …, *s*_*k*_:

$$\begin{array}{c} X^{\prime }\left(t\right)=\sum_{j=1}^{k}L_{s_{j},i_{j}}^{\beta }a_{s_{j},i_{j}}\left(\frac{t-s_{j}}{L_{s_{j},i_{j}}}\right) \end{array}$$ (23)

where $k∼L_{c}^{2-\alpha }$. Applying the Lyaponov Central Limit Theorem condition [45], this implies that *X*′(*t*) converges to Gaussian for large *L*_*c*_ with power-law autocorrelation (see “Central Limit Theorem” in S1 Appendix). Since Gaussian processes are uniquely defined by their second order properties and because a scale-free Gaussian process may be generated by filtering white noise [46], then in analogy to the results for the PF hypothesis $H_{amp}^{\omega }=1/2$ and $E(\rho_{DCCA}\left(n,g_{\omega_{1}}\left(X^{\prime }\left(t\right)\right),g_{\omega_{2}}\left(X^{\prime }\left(t\right)\right)\right))\to 0$ as *n* → ∞.

To derive the Hurst exponent *H*_*raw*_ of *X*′(*t*), we follow [25] and approximate avalanches by a box function:

$$\begin{array}{c} a\left(t\right)=\{\begin{array}{c} 1\;\text{for}\;t\in [0,1]\\ 0\;\text{otherwise} \end{array} \end{array}$$ (24)

The authors show that in this case the autocorrelation function $r\left(t\right)=E\left(X\left(s\right)X\left(s+t\right)\right)-E{\left(X(s)\right)}^{2}$ satisfies:

$$\begin{array}{c} r\left(t\right)\propto \int_{\left|t\right|}^{\infty }\left(L-|t|\right)\int_{0}^{\infty }L^{-\alpha }L^{2\beta }dL \end{array}$$ (25)

$$\begin{array}{c} ∼{\left|t\right|}^{2\beta -\alpha +2} \end{array}$$ (26)

Using the fact that if the autocorrelation function scales as *r*(*t*)∼*t*^−*γ*^, then the Hurst exponent and *γ* are related as *γ* = 2 − 2*H* [47], gives:

$$\begin{array}{c} H_{raw}=\beta -\frac{\alpha }{2}+2 \end{array}$$ (27)

(**MU2**) *α* > 2 and *α* < *β* + 3

For *α* > 2, the probability that an avalanche is active with duration greater than *L*′ is:

$$\begin{array}{c} \frac{1}{T}\sum_{L_{s,I}>L^{\prime }}L_{s,i}∼qE\left(L\;1_{\left(L^{\prime },\infty \right)}\left(L\right)\right) \end{array}$$ (28)

$$\begin{array}{c} ∼\int_{L^{\prime }}^{L_{c}}L^{1-\alpha }dL \end{array}$$ (29)

$$\begin{array}{c} ∼L^{\prime 2-\alpha } \end{array}$$ (30)

The first line follows by definition and the second line is an asymptotic approximation to the first line for large *L*_*c*_. Thus the probability that long avalanches occur simultaneously is negligible; this implies that large avalanches “protrude” from *X*′(*t*) as illustrated in Fig 3.

Moreover, since all quantities take the form of a power-law at criticality [3], we have, for an exponent *β*′, a scaling relation for large *L*:

$$\begin{array}{c} f_{\omega }\left(L^{\beta }a\left(t/L\right)\right)∼L^{\beta^{\prime }}f_{\omega }\left(a\right)\left(t/L\right) \end{array}$$ (31)

Here *f*_*ω*_(*a*)(*t*/*L*) is understood as position *t*/*L* of the normalized process *a*(*t*) filtered in the narrowband around *ω*.

We now derive an expression for *β*′ in terms of the critical exponent *β*. At criticality is has been shown that we require $P\left[\omega \right]∼\omega^{-\beta -1}$ [3]. This implies that the standard deviation of *f*_*Lω*,*L*Δ_(*a*(*t*)) scales according to *L*^−*β*/2^. This is because by definition of the power-spectrum [23]:

$$\begin{array}{c} \text{var}\left(f_{\omega ,\Delta }\left(a\left(t\right)\right)\right)∼\int_{\omega -\Delta }^{\omega +\Delta }P\left[\omega^{\prime }\right]d\omega^{\prime } \end{array}$$ (32)

$$\begin{array}{c} ∼\int_{\omega -\Delta }^{\omega +\Delta }\omega^{-\beta -1}d\omega^{\prime } \end{array}$$ (33)

And therefore:

$$\begin{array}{c} \text{var}\left(f_{L\omega ,L\Delta }\left(a\left(t\right)\right)\right)∼\int_{L\omega -L\Delta }^{L\omega +L\Delta }P\left[\omega^{\prime }\right]d\omega^{\prime } \end{array}$$ (34)

$$\begin{array}{c} ∼\int_{L\omega -L\Delta }^{L\omega +L\Delta }\omega^{\prime -\beta -1}d\omega^{\prime } \end{array}$$ (35)

$$\begin{array}{c} ∼\int_{\omega -\Delta }^{\omega +\Delta }{\left(L\omega^{\prime }\right)}^{-\beta -1}Ld\omega^{\prime } \end{array}$$ (36)

$$\begin{array}{c} ∼L^{-\beta }\text{var}\left(f_{\omega ,\Delta }\left(a\left(t\right)\right)\right) \end{array}$$ (37)

Therefore, for fixed *ω*:

$$\begin{array}{c} \text{std}(f_{L\omega ,L\Delta }\left(a\left(t\right)\right))∼L^{-\beta /2} \end{array}$$ (38)

and:

$$\begin{array}{c} f_{\omega ,\Delta }\left(L_{i}^{\beta }a_{i}\left(t/L_{i}\right)\right)∼L_{i}^{\beta }f_{L_{i}\omega ,L_{i}\Delta }\left(a\right)\left(t/L_{i}\right) \end{array}$$ (39)

$$\begin{array}{c} ∼L_{i}^{\beta /2}f_{\omega ,\Delta }\left(a\right)\left(t/L\right) \end{array}$$ (40)

Therefore by linearity of the Hilbert transform:

$$\begin{array}{c} g_{\omega }\left(L_{i}^{\beta }a_{i}\left(t/L_{i}\right)\right)∼L_{i}^{\beta /2}g_{\omega }\left(a\right)\left(t/L_{i}\right) \end{array}$$ (41)

Thus:

$$\begin{array}{c} \beta^{\prime }=\beta /2 \end{array}$$ (42)

**Fig 12:**
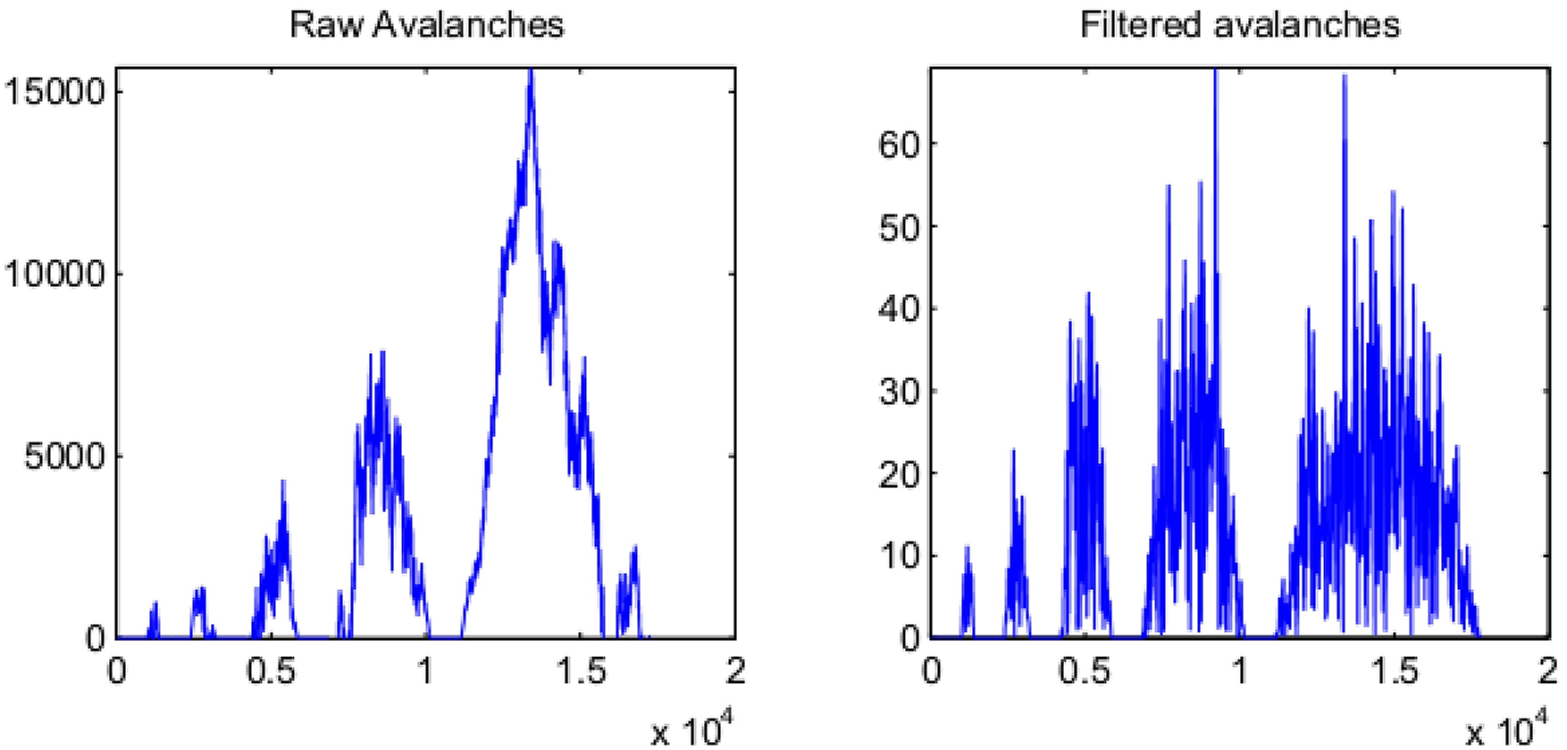
The difference in scaling between the raw avalanches and their filtered amplitudes. In this figure we set *β* = 1. The left hand panel displays avalanches *L*^*β*^ *a*(*t*/*L*) with log-spaced lifetimes *L* between 400 and 7000. The right hand panel displays the narrowband amplitudes of these avalanches *L*^*β*′^ *g*_*ω*_(*a*)(*t*/*L*). Since *β* > *β*′ = *β*/2 we see that the narrowband amplitudes scale less steeply than the raw avalanches.

An illustration of the relationship between *β* and *β*′ is given in Fig 12; if the height of avalanches scales with their length (left panel), then the scaling of the height of their narrowband amplitude envelopes is less sharp (right panel). We validate this relation in “Further Simulations: Exponent Relations” in S1 Appendix.

We now require an additional known relation between critical exponents. Let *S* be the size of an avalanche, i.e. the total activity occurring over the course of the avalanche: $S=\int_{0}^{L}L^{\beta }a\left(t/L\right)dt$. Then, it is known that at criticality [3]:

$$\begin{array}{c} S∼S^{-\tau } \end{array}$$ (43)

And [2]:

$$\begin{array}{c} \frac{\alpha -1}{\tau -1}=\beta +1 \end{array}$$ (44)

The “filtered” avalanches $g_{\omega }\left(L_{s,i}^{\beta }a\left(t/L_{s,i}\right)\right)$ also obey power-laws, with *different* exponents, *α*′, *β*′, *τ*′. We showed above that *β*′ = *β*/2; in addition, *α*′ = *α* since $g_{\omega }\left(L_{s,i}^{\beta }a\left(t/L_{s,i}\right)\right)$ and $L_{s,I}^{\beta }a\left(t/L_{s,i}\right)$ have the same length.

By Eq (44):

$$\begin{array}{c} \frac{\alpha^{\prime }-1}{\tau^{\prime }-1}=\beta^{\prime }+1 \end{array}$$ (45)

The size exponents *τ* and *τ*′ are important for our subsequent analyses because whether the asymptotic properties of the process *X*′(*t*) are dominated by the large avalanches depends on whether their size distributions have divergent variance or not. See Fig A in S1 Appendix for an illustration of these “filtered avalanches”. *X*′(*t*) may be divided into a sum of long avalanches *L*_*s*,*i*_ > *L*′ which do not overlap and short avalanches.

$$\begin{array}{c} X^{\prime }\left(t\right)=\sum_{L_{s,i}>L^{\prime }}L_{s,i}^{\beta }a_{s,i}\left(\frac{t-s}{L_{s,i}}\right)+\sum_{L_{s,i}\leq L^{\prime }}L_{s,i}^{\beta }a_{s,i}\left(\frac{t-s}{L_{s,i}}\right) \end{array}$$ (46)

Therefore, since *f*_*ω*_(*L*^*β*^)∼*L*^*β*/2^ *f*_*ω*_(*a*)(*t*/*L*):

$$\begin{array}{ccc} f_{\omega }\left(X^{\prime }\left(t\right)\right) & ∼ & \sum_{L_{s,i}>L^{\prime }}L_{s,i}^{\beta /2}f_{\omega }\left(a_{s,i}\right)\left(\frac{t-s}{L_{s,i}}\right)\\ & & +\sum_{L_{s,i}\leq L^{\prime }}L_{s,i}^{\beta /2}f_{\omega }\left(a_{s,i}\right)\left(\frac{t-s}{L_{s,i}}\right) \end{array}$$ (47)

The left hand term dominates whenever its variance is unbounded for large *L*_*c*_. This happens if *τ*′ < 3 (since then the variance of *p*(*S*) diverges) which translates to *α* < *β* + 3 by Eq (45). Therefore the right hand term may be neglected and since the avalanches of the left hand term are separated in time, the envelope operator may be pulled under the sum so that:

$$\begin{array}{c} g_{\omega }\left(X^{\prime }\left(t\right)\right)∼\sum_{L_{s,i}>L}L_{s,i}^{\beta /2}g_{\omega }\left(a_{s,i}\right)\left(\frac{t-s}{L_{s,i}}\right) \end{array}$$ (48)

The same proof as for *H*_*raw*_ when *α* < 2 may then be applied to deriving $H_{amp}^{\omega }$. The same proof applies for *H*_*raw*_ as before.

Thus we find:

$$\begin{array}{ccc} H_{amp}^{\omega } & = & \beta /2-\alpha /2+2\\ & > & 1/2 \end{array}$$ (49)

And:

$$\begin{array}{ccc} H_{raw}^{\omega } & = & \beta -\alpha /2+2\\ & > & 1/2 \end{array}$$ (50)

Moreover, assuming that the integrals $\int_{0}^{1}g_{\omega }\left(a\left(t\right)\right)dt$ exist, then the separation of large avalanches make it simple to derive that *ρ*_*DCCA*_(*n*, *ω*_1_, *ω*_2_) → 1 as *n* → ∞: for large *n*, each DCCA window is longer than the largest avalanche. For *α* < *β* + 3 the avalanches in the left hand term of Eq (47) may be assumed to be non-overlapping since we assume *α* > 2 (see derivation for MU1). Because *τ*′ < 3, we may neglect small avalanches and assume that a window contains only one avalanche at *t*′ with length *L*. Then:

$$\begin{array}{c} \sum_{i=1}^{t}g_{\omega_{j}}\left(X\right)\left(i\right)∼\{\begin{array}{cc} 0 & \;\text{if}\;t<t^{\prime }\\ L^{\beta /2}\int_{0}^{L}g_{\omega_{j}}\left(a\left(t/L\right)\right) & \;\text{if}\;t>t^{\prime }+L \end{array} \end{array}$$ (51)

But $\int_{0}^{L}g_{\omega_{1}}\left(a\left(t/L\right)\right)∼\int_{0}^{L}g_{\omega_{2}}\left(a\left(t/L\right)\right)$ up to a constant factor for large avalanches and assuming the integral converges. Thus the correlation tends to 1.

(**MU3**) *α* > 2 and *α* < 2*β* + 3

For frequencies with 1/*ω* ≫ *L*_*c*_ and *L* ≤ *L*_*c*_:

$$\begin{array}{c} f_{\omega }\left(L^{\beta }\right)∼L^{\beta }f_{\omega }\left(a\right)\left(t/L\right) \end{array}$$ (52)

This is because relative to the time-scale of the filter, *a*(*t*/*L*) may be treated as a delta function, which weights all frequencies equally; *y*(*ω*)∼*c*, where *y*(*ω*) is the power-spectrum of *a*(*t*/*L*) and *c* is a constant. Therefore we apply the same argument as for MU2 but with Eq (47) replaced by:

$$\begin{array}{ccc} f_{\omega }\left(X^{\prime }\left(t\right)\right) & ∼ & \sum_{L_{s,i}>L}L_{s,i}^{\beta }f_{\omega }\left(a_{s,i}\right)\left(\frac{t-s}{L_{s,i}}\right)\\ & & +\sum_{L_{s,i}\leq L}L_{s,i}^{\beta }f_{\omega }\left(a_{s,i}\right)\left(\frac{t-s}{L_{s,i}}\right) \end{array}$$ (53)

For *τ* > 3, the left hand term dominates, since it has unbounded variance in *L*_*c*_, so we find that for *ω*_1_, *ω*_2_ → 0, *ρ*_*DCCA*_(*n*, *ω*_1_, *ω*_2_) → 1 as *n* → ∞. Moreover, applying the results for MU2 we have:

$$\begin{array}{c} H_{amp}^{\omega }=1/2 \end{array}$$ (54)

$$\begin{array}{c} H_{raw}^{\omega }=\beta -\alpha /2+2 \end{array}$$ (55)

$$\begin{array}{c} >1/2 \end{array}$$ (56)

(**MU4**) *α* > 2*β* + 3 and *α* > 2

Since these universality classes have the shortest tails in their critical distributions, we may apply identical methods as applied to the PF hypothesis which show that $H_{amp}^{\omega }=H_{raw}=1/2$ and *ρ*_*DCCA*_(*n*, *ω*_1_, *ω*_2_) → 0 as *n* → ∞ when *ω*_1_ ≠ *ω*_2_.

### Simulation details

In all simulations involving CD we model the average avalanche shape of Eq (9) as a quadratic function: *b*(*t*) = −4(*t* − 1/2)^2^ + 1 and we set *b*(*t*) = *c*(*t*). For the noise component we use the implementation of [30]. For the power-law cutoff sampling, we perform a density transformation of the uniform distribution. (In MATLAB x = rand(1,T).*(1-*L*_*c*_)+*L*_*c*_; x = 1./(x. ^ (1./(*α*-1))))

For the PF model since the distribution of bursts of activity according to the PF decays quickly, we take *X*(*t*) as a Gaussian white noise process. *X*′(*t*) is then obtained by filtering using the method of [30] to yield a process with spectrum scaling according to 1/*ω*^*γ*^.

### Experiment

Seven subjects participated in the study (1 female); the subjects had an average age of 25 years at the time of the study. Participants gave written informed consent for their participation. The experimental protocol was approved by the Institutional Review Board of the Charité Medical University, Berlin and conformed to the declaration of Helsinki. EEG recordings were obtained at rest with subjects seated comfortably in a chair with their eyes open. Recordings were made of three sessions, each 5 minutes long so that each data set comprises roughly 15 minutes of data. EEG data were recorded with 96 Ag/AgCl electrodes, using BrainAmp amplifiers and BrainVision Recorder software (Brain Products GmbH, Munich, Germany). The signals were recorded in the 0.016–250 Hz frequency range at a 1000Hz sampling frequency and subsequently subsampled to 200Hz.

Outlier channels were rejected after visual inspection for abrupt shifts in voltage and poor signal quality. The data were then re-referenced according to the common average. Spatial filters were computed on the data using Spatio-Spectral Decomposition (SSD) [48], in order to extract components with pronounced alpha oscillations. Spatial filters with poor signal quality or topographies were rejected. We then restricted our analysis to components displaying a peak in the alpha range; this step ensured a high signal quality with low levels of artifactual activity. The fact that the spatial filters yield clear oscillatory signals ensured that the neuronal processes in the adjacent frequency ranges similarly originated from cortical areas relating to neuronal rather than artifactual activity. For DFA and DCCA estimation we set *n* to log-spaced values between 1000 and 25000.

Important is that we analyse 3 frequency ranges without oscillations (no local maximum in power-spectrum); the aim was to restrict analysis to activity corresponding to the 1/*f*^*γ*^ shape of the power-spectra. Given that the data were sampled at 200Hz, and that lower frequencies require far larger window sizes for analysis, we chose 3 frequencies above the beta range, taking care to exclude the 50Hz line noise.

## Supporting information

S1 Appendix Supplementary theory, simulations and data analysis. Proof of the central limit theorem discussed in Section: Materials and Methods: Theory: Predictions for the CD Theory; further simulations validating the theoretical exponent relations displayed in Table 1 and the effect of signal-to-noise ratio; further data analysis investigating the effect of the choice of spatial filter, power spectra and DFA log-log plots.
(PDF)

S1 Software CD model code. Fast implementation of the CD model of Eq (1).
(GZ) Click here for additional data file.
