# Supplementary material for: Long-range temporal correlations in neural narrowband time-series arise due to critical dynamics
Source: PLoS One. 2017 May 4;12(5):e0175628. doi: 10.1371/journal.pone.0175628 (PMC5417502; doi:10.1371/journal.pone.0175628)
Supplement: S1 Appendix — Proof of the central limit theorem discussed in Section: Materials and Methods: Theory: Predictions for the CD Theory; further simulations validating the theoretical exponent relations displayed in Table 1 and the effect of signal-to-noise ratio; further data analysis investigating the effect of the choice of spatial filter, power spectra and DFA log-log plots. (PDF) [file pone.0175628.s001.pdf]

S1 Appendix: Long-range temporal correlations in neural narrowband time-series arise due to critical dynamics

April 18, 2017

Central Limit Theorem for $\alpha < 2$

At any time point we have a number $k \sim L_c^{2-\alpha}$ of avalanches which commenced each at times $s_i = 1, \ldots, k$ with lengths L_i. Then we may write:

$$X(t) = \sum_{i=1}^{k} L_i^{\beta} a\left(\frac{t - s_i}{L_i}\right) \quad (1)$$

For k large, after normalization and centering we apply the Lyapunov central limit condition [45] to:

$$\bar{X}(t) = \frac{1}{\sqrt{k} L_c^{\beta+(\alpha+1)/2}} \sum_{j=1}^{k} L_i^{\beta} \left(a\left(\frac{t - s_i}{L_i}\right) - \mu\right) \quad (2)$$

where μ is the mean value of $a(t)$ over the interval $[0, 1]$. Let $s_k^2 = kL_c^{2\beta+\alpha+1}$, then for the Lyapunov condition to apply we need to show, for some $\delta > 0$, that:

$$\frac{1}{s_k^{2+\delta}} \sum_{j=1}^{k} \mathbb{E} \left| L_i^{\beta} \left(a\left(\frac{t - s_i}{L_i}\right) - \mu\right)\right|^{2+\delta} \to 0 \quad (3)$$

Thus:

$$\frac{1}{s_k^{2+\delta}} \sum_{j=1}^{k} \mathbb{E} \left| L_i^{\beta} \left(a \left(\frac{t - s_i}{L_i} \right) - \mu \right) \right|^{2+\delta} \tag{4}$$

$$\sim \frac{1}{k^{1+\delta/2} L_c^{(\beta+\alpha/2+1/2)(2+\delta)}} \sum_{i=1}^{k} L_i^{(2+\delta)/\beta} \tag{5}$$

$$\sim \frac{1}{k^{1+\delta/2} L_c^{(\beta+\alpha/2+1/2)(2+\delta)}} k L_c^{\beta(2+\delta)+\alpha+1} \tag{6}$$

$$\sim \frac{L_c^{\alpha+1}}{k^{\delta/2} L_c^{(\alpha/2+1/2)(2+\delta)}} \tag{7}$$

$$\leq \frac{1}{k^{\delta/2} L_c^{\delta/2}} \to 0 \tag{8}$$

This completes the proof for univariate Gaussianity. The proof for the Gaussian process is a simple extension by the Cramer-Wold device [42] and the observation that the avalanches have bounded length.

1 Further Simulations

1.1 Exponent Relations

The aim of the first simulation in this section is to verify the prediction that $\beta' = \beta/2$, Equation (40) of the main paper, by measuring the heights of the filtered avalanches $a_\omega(t)$. We set $\beta = 0.25$, $L = 2^{10}, \dots, 2^{13}$ and for each L considered, we simulate 10^4 avalanches of this length and calculate the mean avalanche profile. We then log-regress the height of these profiles against $\log(L)$.

The results are displayed in Fig. A. Close agreement is observed between the theoretical estimate $\beta' = \beta/2$ and the simulated results; the prediction improves for higher β.

In the second simulation we check the expressions for H_{amp} and H_{raw} in terms of α and β, Equations (47) and (48) of the main paper, seting $\alpha = 2.5$, $q = 1$, $L_c = 10^6$, with a burn in time of 10^5 time points, setting n to log spaced values between 100 and 7000 for the estimation of H_{raw} (according to where scaling regions were observed). The results are displayed in Fig. B.

The quality of the H_{raw} estimate is greater for small β, whereas the quality of the H_{amp} estimate is greater for larger β. This discrepancy may be explained as follows: since $X(t)$ has longer tails than $g_\omega(X(t))$, the convergence of its empirical moments is slow for large β thus the quality of the estimate decreases for large β. On the other hand, the estimate of H_{raw} requires a linear approximation to the non-linear transform given by the amplitude of the analytic signal: this approximation increases in quality for larger β.

1.2 The Effect of Signal-to-Noise Ratio

We now consider a potential limitation of our techniques. In analysis of neural dat, such as EEG or MEG data, the brain signal is never measured in isolation but rather as a linear mixture with noise sources. Typically these noise sources are assumed to be uncorrelated white noise [34, 48]. We now investigate the effect such noise sources might have on the values of $\rho_{DCCA}(n)$ for the CD model. Since white noise has $\rho_{DCCA}(n) = 0$, adding white noise to the PF model measurements will not affect these greatly. (The effect o noise on the estimation of Hurst exponents was already considered by [34] who find that for true exponents greater than 1/2, the measured exponents are smaller than in the absencen of noise.) To this end we generate 20 signals from the CD model, Equation (1) of the main paper, in MU2 with $\alpha = 2.5$ and $\beta = 1, q = 5$ and $T = 40000$. We add uncorrelated Gaussian white noise at signal-to-ratios of 100, 10 and 1 (measured as ratios between the variances of the signals) and then measure $\rho_{DCCA}(n, \omega_1, \omega_2)$, for $\omega_1 = 0.6$ and $\omega_2 = 0.8$ as a fraction of the Nyquist frequency, with $\Delta = 0.04$ and n log-spaced between 140 and 23000.

The results are displayed in Fig. C. We see that at higher SNR values $\rho_{DCCA}(n)$ is indeed close to 1 at large scales, as predicted by our theory for MU2, but at medium to low SNR, we find that $\rho_{DCCA}(n)$ is significantly less than 1. We find, nevertheless, that $\rho_{DCCA}(n)$ continues to increase to high scales.

2 Further Data Analysis

In this section we repeat the analysis of the real data reported in main paper, but with a different choice of spatial filter. Instead of taking SSD filters, which the signal-to-noise ratio in the alpha range to obtain filters corresponding to neuronal activity, we take Laplacian spatial filters [20], which act as spatial high-pass filter. All other paramters remain fixed.

The results are displayed in Fig. D and confirm that the results obtained on real data using the SSD filters calibrated to the alpha rhythm may also be obtained using filters which do not depend on the alpha rhythm.

To further check that results do not depend on the detrending degree d, we repeat this analysis with $d = 2$. The results are displayed in Fig. E and show qualitatively identical trends; most notable is that the correlation is less noisy for higher degree detrending, which we conjecture is due to a more thorough removal of trends.

We also compute power-spectra of the first SSD component for each of the 7 subjects, using Welch's method (Hanning window of length 2 seconds, with 0 overlap between windows and calculated at 256 points). The spectra are displayed in Fig. F; each spectrum clearly displays the presence of alpha/ mu range oscillations at around 10Hz. Note, however, that all analysis was performed for frequency bands located outside the peaky parts of the power-spectra.

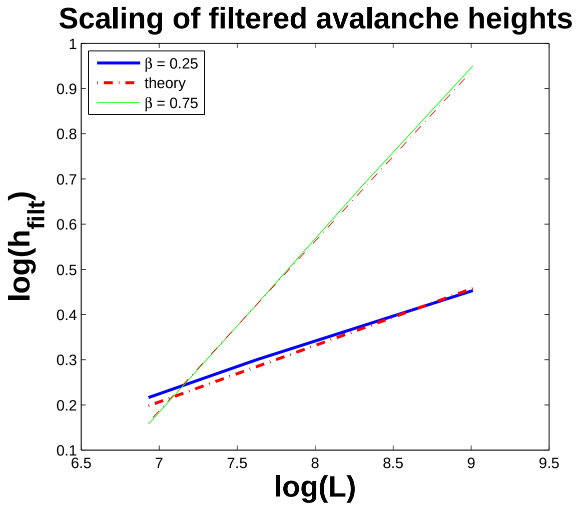

Figure A: Comparison of the estimate $\beta' = \beta/2$ for the scaling of heights of filtered avalanches vs. simulation. See Equation (40) of the main paper. Details of the simulation are given in S1 Appendix: Further Simulations: Exponent Relations.

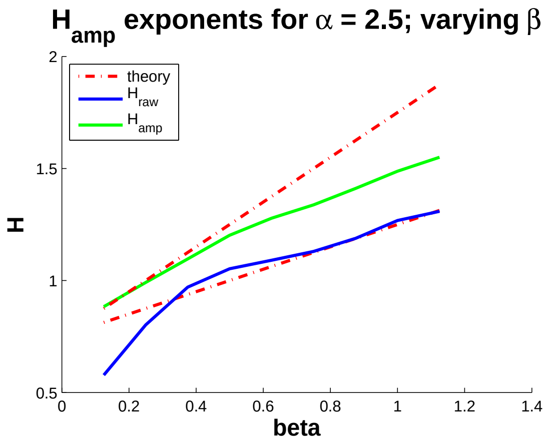

Figure B: Scaling of H_{amp} and H_{raw} in simulation compared to theory. The theoretical estimate is $H_{amp} \sim 2 - \alpha/2 + \beta'$ for $\beta' = 1$. See S1 Appendix: Further Simulations: Exponent Relations for details of the simulation

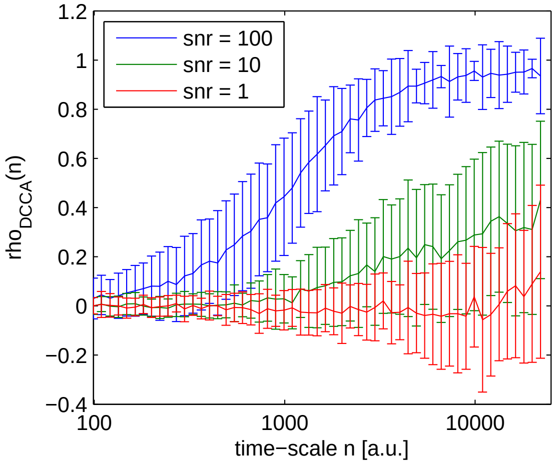

Figure C: The figure displays $\rho_{DCCA}(n)$ values in MU2 for a range of signal-to-noise ratios. See S1 Appendix: Further Simulations: The Effect of Signal-to-Noise Ratio for details of the simulation.

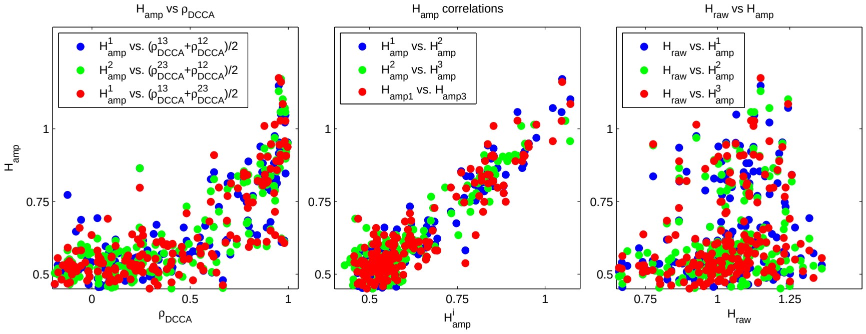

Figure D: The figure displays results in an identical analysis as carried out for Fig 9 of the main paper, with the only difference being that we use Laplacian rather then SSD spatial filters. See S1 Appendix: Further Data Analysis.

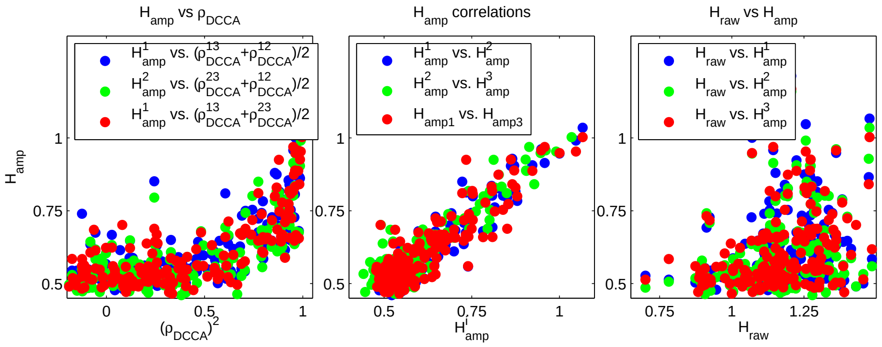

Figure E: The figure displays results in an identical analysis as carried out for Fig 9 of the main paper, with the difference being that we use Laplacian rather then SSD spatial filters and quadratic detrending with d = 2. See S1 Appendix: Further Data Analysis.

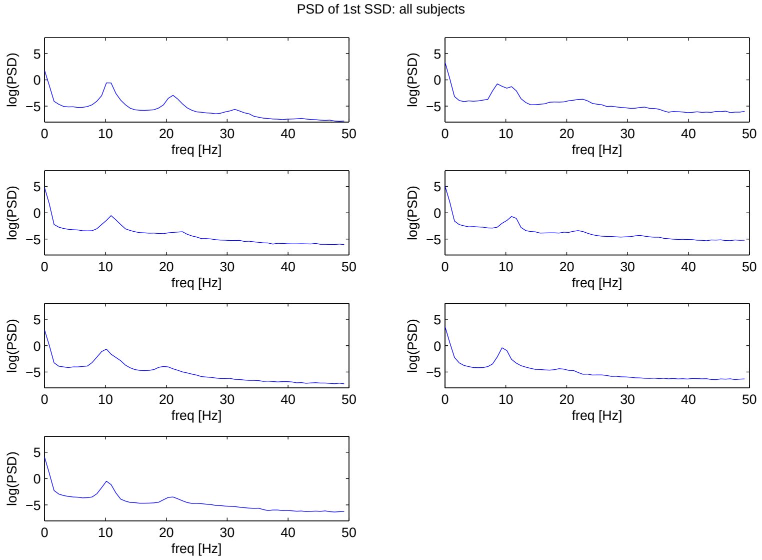

Figure F: The figure displays power-spectra from each of the 7 subjects considered in the study. In each case the power-spectrum is estimated on the first SSD component using Welchs method. See S1 Appendix: Further Data Analysis.
